# Scaling up mechanochemical reactions: linking crystalline phase evolution studied *via in situ* PXRD with kinetics from MCR-ALS

**DOI:** 10.1039/d6sc01158f

**Published:** 2026-05-12

**Authors:** Laura Macchietti, Maria Carta, Francesco Delogu, Fabrizia Grepioni, Franziska Emmerling, Lucia Casali

**Affiliations:** a Department of Chemistry ‘Giacomo Ciamician’, University of Bologna Via Gobetti 85 Bologna 40129 Italy; b Department of Mechanical, Chemical and Materials Engineering, University of Cagliari Via Marengo 2 Cagliari 09123 Italy; c Center for Colloid and Surface Science (CSGI), Cagliari Research Unit, Department of Chemistry, University of Florence Via Della Lastruccia 3 Sesto Fiorentino 50019 FI Italy; d Federal Institute for Materials Research and Testing Richard-Willstätter-Straße 11 Berlin 12489 Germany lucia.casali@bam.de; e Department of Chemistry, Humboldt-Universität zu Berlin Berlin 12489 Germany

## Abstract

This work addresses a key challenge in scaling up mechanochemical synthesis: deriving a kinetic model when unpredictable formation and intricate interaction of multiple crystalline phases occur during solid-state transformations. Reaction kinetics translate our understanding of chemical processes into mathematical rate expressions used for reactor design and evaluation, thus representing a challenge to be addressed for the scale up at the industrial level. Choosing co-crystallization of chloro-3-sulfamoylbenzoic acid (CSBA) and isonicotinamide (INA) as a model system, at first we employ time-resolved *in situ* powder X-ray diffraction (PXRD) and multivariate curve resolution-Alternating Least Squares (MCR-ALS) analysis to quantify and resolve the evolution of crystalline intermediates under varying methanol-assisted conditions. Our data show that even small changes in the amount of methanol can dramatically alter the kinetic profile, stabilise transient phases (including some that were previously unreported) and alter the overall reaction pathway. We then demonstrate the robust deconvolution of overlapping phases and the extraction of quantitative rate parameters that rationalize the observed behaviour by integrating kinetic modelling as a soft-hard constraint in the MCR-ALS workflow. The validation of the established MCR-ALS workflow is achieved by applying a phenomenological kinetic modelling tailored to rationalize the mechanochemical reaction rates. These results establish a broadly applicable platform for analysing and controlling the complex phase evolution, along with the derivation of a kinetic model instrumental to mechanochemical process development and scaling up, thereby supporting the transition of sustainable solid-state syntheses from the laboratory to industry.

## Abstract 2

The proposed *in situ* PXRD/MCR-ALS platform resolves complex phase evolution and derives kinetic models to advance mechanochemical scale up.

## Graphical abstract

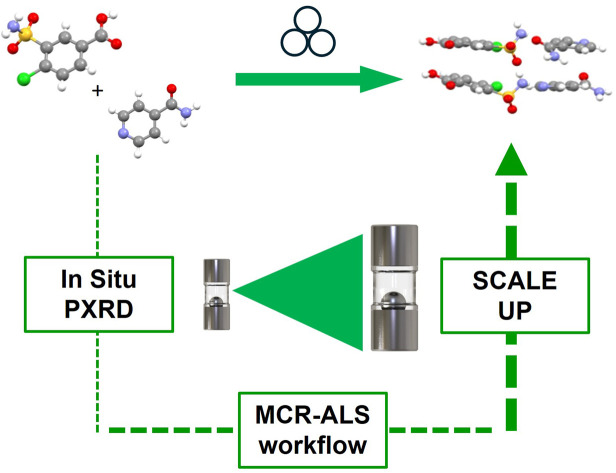

## Introduction

Growing environmental and regulatory pressures are exposing the economic and ecological limitations of traditional solution-based chemical manufacturing, particularly in terms of solvent consumption and waste generation.^1^ Mechanochemistry offers a solvent-free or solvent-minimized alternative that can reduce waste, improve atom economy and align with the UN Sustainable Development Goals.^2–4^ In fact, being a solvent-free method which drives the chemical reactions *via* input of mechanical energy,^5,6^ mechanochemistry is attracting the interest of industries that are increasingly looking to invest in sustainable and cost-effective chemical technologies.^7–9^

Scaling up mechanochemical reactions remains challenging,^10–12^ mainly because of the limited understanding of solid-state transformation mechanisms and their frequent deviation from the kinetic and thermodynamic principles established for solution chemistry.^13^ This is widely recognized as a key challenge when adapting mechanochemical processes for industrial use, while solution-based reactions benefit from well-established scaling strategies and mechanistic models.^14,15^ Consequently, the mechanochemical community is developing methods and strategies aimed to gain control over the mechanochemical reactions.^16,17^ A recent approach consisted in the development of a freely accessible online tool for calculating energy parameters across different planetary and mixer mills, thereby facilitating the reproducibility of mechanochemical reactions across the most commonly used laboratory mills.^18^ The continued development and application of time-resolved *in situ* (TRIS) techniques for monitoring mechanochemical reactions offered, instead, an advanced tool for elucidating the reaction mechanism.^19–21^

Above all, the derivation of reaction kinetics represents a crucial step for advancing the scale up of mechanochemical reactions.^22^ Reaction kinetics translate our understanding of chemical processes into mathematical rate expressions used for reactor design and evaluation.^23^ Within the industrial context, it does imply that kinetic models are being implemented in process simulators in order to thoroughly design and optimize chemical processes.^24^ Derivation of reaction kinetics and developing of kinetic models are therefore priority tasks, which have been and still are addressed in several fields of chemistry.^25,26^

Among the approaches to obtain kinetic information from *in situ* data suitable for kinetic modeling, we demonstrated in an earlier study that the MCR-ALS method can be used to obtain kinetic information from PXRD data by comparing it with Rietveld refinement.^27,28^ Although MCR-ALS has already been applied to *in situ* spectroscopic data,^29,30^ the use of chemometrics with PXRD data for quantitative information remains uncommon and enforces the novelty of this approach,^31–33^*i.e.* the direct extraction of kinetic equations from PXRD data.

**Fig. 1:**
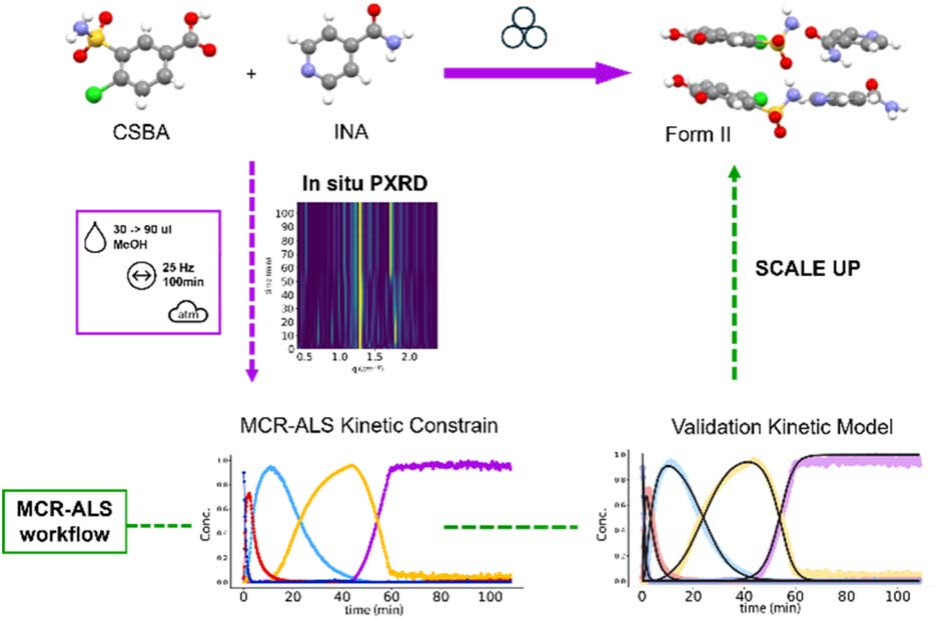
Schematic representation of the MCR-ALS workflow (green arrow) for the scale-up of the mechanochemical synthesis of Form II (violet arrows).

In this work we investigated the co-crystallization of chloro-3-sulfamoylbenzoic acid (CSBA) and isonicotinamide (INA) as a benchmark system to evaluate the robustness of a MCR-ALS workflow for the scale up of mechanochemical reactions (Fig. 1).^34^ The compounds investigated are known to form co-crystals with different stoichiometries, including 1 : 1 (Form I, FI; Form II, FII), 2 : 1 (CC_21), and 1 : 2 (CC_12) ratios (see SI 1),^35,36^ depending on the milling conditions.

Using the MCR-ALS approach,^37^ we were able to (i) resolve the complex interplay between multiple crystalline phases, (ii) identify and quantify a previously unknown compound, and (iii) extract kinetic and mechanistic information from this intricate dataset.

By integrating kinetic modelling as soft-hard constraint in the MCR-ALS workflow, we improved the deconvolution and quantification of overlapping phases, along with a rationalization of the observed kinetics.^38^ The validation of the established MCR-ALS workflow was finally achieved by applying a phenomenological kinetic modelling tailored to rationalize the mechanochemical reaction rates.^39^

In conclusion, this work demonstrates the robustness of the chemometric approach MCR-ALS to X-ray diffraction data, thus highlighting the versatility of the method.^40^ It also provides a proof of concept for how combining methodologies from different areas of chemistry can substantially advance and enrich mechanochemical research.

## Results and discussion

### *In situ* data

The co-crystallization of chloro-3-sulfamoylbenzoic acid (CSBA) and isonicotinamide (INA), yielding the 1 : 1 cocrystal Form II was systematically investigated by varying the amount of solvent added during milling,^41^ respectively l30, 60 and 90 µL of MeOH (see SI 2–3). In fact, the amount of solvent has already been proven to play a pivotal role in determining both the kinetics of the transformations and the stability of potential intermediate phases, thus influencing the reaction pathway.^42^ With respect to the choice of the solvent, we explored several of the liquid additives screened in the mechanochemical co-crystallizations.^34^ Among them, MeOH proved to be the most suitable choice, as it led to the most complex and informative phase evolution, making it particularly appropriate for testing our methodology.

**Fig. 2:**
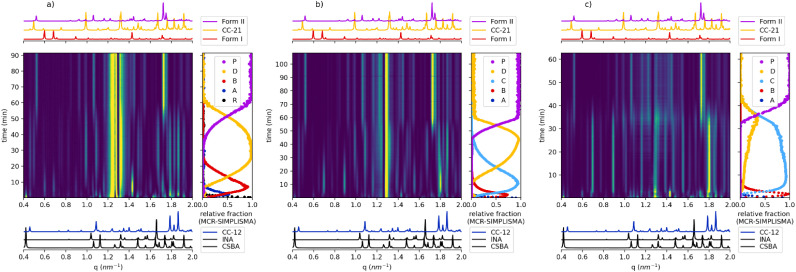
2D plots of the PXRD evolution over time for (a) 30 µL system (left), (b) 60 µL system (middle) and (c) 90 µL system (right) along with the kinetic data derived from the MCR-ALS SIMPLISMA method (R, A, B, D, P abbreviations are explained in the MCR-ALS method section).

At first, we investigated the reaction with the lowest content of MeOH. As shown in Fig. 2a, the reaction displayed a highly complex and dynamic evolution. During the first 5 minutes of milling, the diffraction data indicated the presence of the unreacted starting materials. Between 5 and 15 minutes, these reagents gradually transformed into Form I, which represented the first crystalline product detected under these conditions. In the time window between 15 and 60 minutes, both CC_21 and CC_12 were observed simultaneously, and finally the reaction reached completion, yielding exclusively Form II as the final and most stable product.

When the MeOH content was increased up to 60 µL, the reaction pathway changed significantly (Fig. 2b). The formation of Form I was much less pronounced and was only observed during the first few minutes of milling. At the same time, a new and previously unreported crystalline phase appeared and persisted for approximately 30 minutes before converting into a physical mixture of CC_21 and CC_12. The stabilization of this transient phase, possibly corresponding to a solvate or a new polymorphic form, highlights the influence of solvent quantity on the reaction pathway and suggests that even small changes in solvent content can alter the mechanochemical process.

Finally, further increasing the solvent amount up to 90 µL let to an even faster progression of the reaction (Fig. 2c). In this case, the lifetime of both CC_21 and CC_12 was significantly shortened, and the conversion to Form II occurred roughly 20 minutes earlier compared to the systems with lower MeOH content.

Overall, the comparative analysis of these reactions clearly demonstrates that the amount of MeOH influences the mechanochemical reaction pathway between INA and CSBA. Increasing the solvent quantity systematically led to (i) destabilization and reduced persistence of Form I, (ii) formation and temporary stabilization of the new unknown phase, (iii) destabilization of the CC_21 and CC_12 mixture, and (iv) accelerated appearance of the final Form II.

These findings underscore the complex and highly dynamic nature of liquid-assisted mechanochemical processes, in which the solvent does not merely act as a medium but also modulates the reaction kinetics and phase stabilities.^43^

### MCR-ALS method

Multivariate Curve Resolution (MCR) is a soft-modelling approach that decomposes a data matrix into concentration and pure component profiles under a bilinear model. The method exploits data variability to resolve the contributions of mixture components, while appropriate constraints are required to obtain chemically meaningful and interpretable solutions (see SI 4). We have previously shown that the MCR-ALS approach can be applied to PXRD data, providing quantitative results comparable to traditional Rietveld refinement.^27^

Within this framework, the application of the MCR-ALS approach to the three datasets demonstrates the robustness and reliability of the method for the analysis of PXRD data, while minor resolution limits are discussed as the systems increase in complexity (see SI 4). In the first case, several crystalline phases were observed, which the method successfully detected and fully resolved, as the acquisition rate was well matched to the kinetics under investigation. In the second dataset an additional unknown phase was identified, which the method successfully isolated and quantitatively described, without the need for any reference pattern. In the third and most challenging system, the reaction involved the formation of the unknown phase, along with considerably shorter intermediate lifetimes, that tested the deconvolution capability. Overall, the method identified and quantified all crystalline components present (see all Fig. SI8). The results show that by applying the simplest implementation it was possible to gather useful insights into the species evolution.

The minimal requirement for executing MCR-ALS is an initial estimate of either the concentration matrix (*C*_0_) or the pure profile matrix (*S*_0_) to initiate the iterative process and define the number of components for the deconvolution. In our previous work,^27^ the SIMPLISMA-based estimation of *S*_0_ was identified as an effective approach for PXRD signal deconvolution, owing to the sharp diffraction peaks that facilitate the purest variable selection method. In the present study, the method was able to detect and separate five distinct components for all the datasets, summarized in Table 1.

**Table 1:** Main phases identified for each component of the MCR-ALS (SIMPLISMA) calculation. Abbreviations for the phases are also reported for clarity

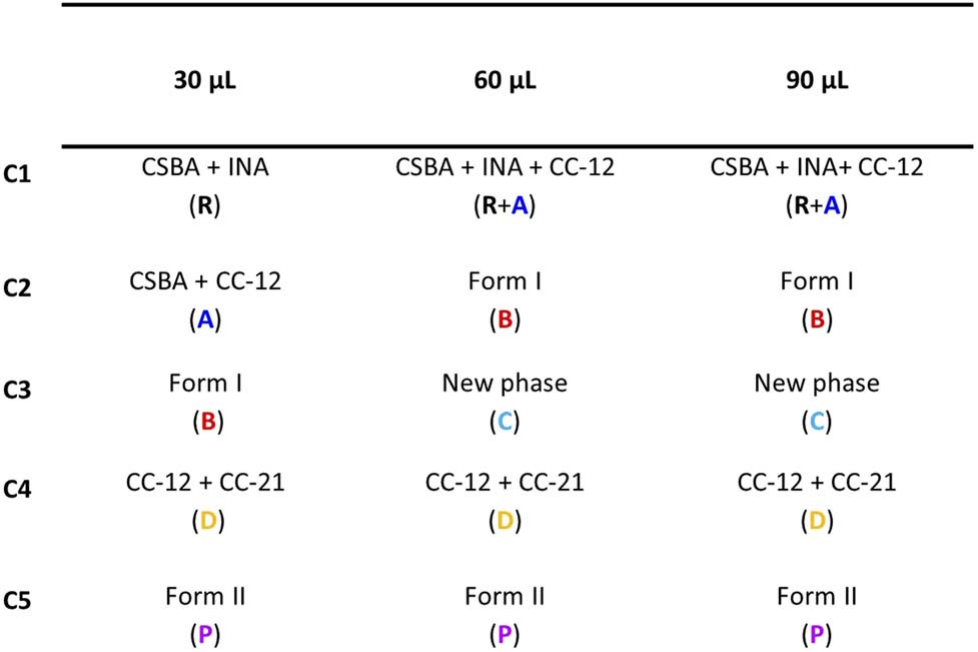

The inspection of the components listed in Table 1 shows that the CC_21 and CC_12 mixture consistently appears as the final intermediate under all conditions, despite the emergence of a new phase at high MeOH content. CC_12 forms early during milling as the product of the rapid reaction between INA and part of CSBA, being then the first intermediate prior to Form I.

The phase attribution confirms this sequence: in the 30 µL experiment, it constitutes a well-defined transition represented by two distinct components (C1–C2, Fig. SI.8.1). At higher solvent volumes (60 and 90 µL), INA consumption and CC_12 formation occur in less than one minute. This leads the algorithm to merge reagents and intermediate into a single component (C1, Fig. SI.8.2–3), as the rapid changes are insufficiently captured by the adopted sampling strategy. Consequently, the limited variability in the data induces rank deficiency in the matrix (see SI 4), hindering component separation. Rank deficiency represents a major limitation for MCR, as it directly affects both resolution capability and quantitative accuracy, and must therefore be carefully considered. The overlap involving the reactants and CC_21 affects the deconvolution of the first component at 60 µL and 90 µL. However, the resulting combined contribution can still be rationalized as a single reaction step (C1, Table 1), similarly to the grouping of reagents, both arising from rank deficiency effects. While this condition limits the detailed quantification of individual phases, treating reactant species as a combined contribution was considered an acceptable simplification for the purpose of kinetic evaluation.

**Fig. 3:**
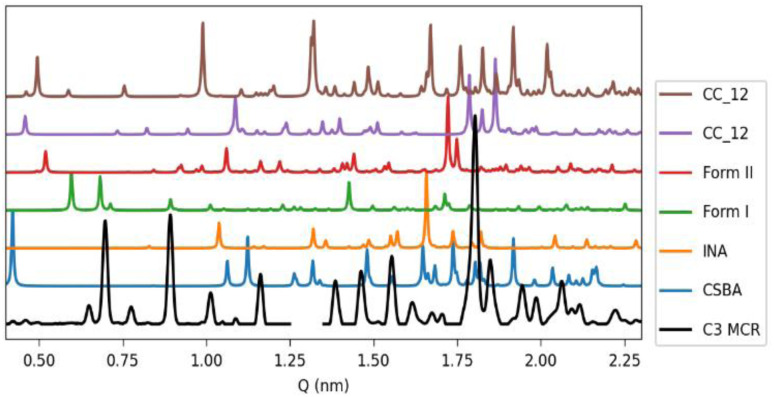
Pure profile of component C3 from MCR-ALS analysis of the 90 µL experiment (black), compared with calculated patterns from single crystal structures of the known phases of the system.

The exploratory analysis clarified the main reaction transitions, providing preliminary concentration profiles that represent the complex dynamics of the system (Fig. 1). Additionally, the decomposition yielded a distinct profile for the new phase observed under high solvent conditions, which can serve as a reference for future studies. Comparison of this extracted profile with the reference pattern of the known phases confirms its unique character (Fig. 3).

The analysis of the pure profiles indicates an overall satisfactory decomposition, confirming the robustness of the method in systems with multiple components and validating the phase assignments. However, mixed profiles were primarily observed for component two (C2) in both the 60 and 90 µL LAG experiments, corresponding to Form I, and for component four (C4) in the 90 µL experiment, associated with the CC_21 and CC_12 mixture (Fig. SI 8.2–3). In such cases, the mixed profiles must be evaluated in relation to the scope of the analysis. Minor “contaminant” contributions can be neglected for kinetic purposes if the main component is clearly identified, as their impact on the overall profile is minimal. Conversely, when external contributions are dominant, as in component four (C4) in the 90 µL experiment, the quantitative reliability is significantly compromised. Nevertheless, the analysis still captures the qualitative behaviour of the reaction, highlighting the slow kinetics of the CC_12 and CC_21 mixture. Notably, this information was used as starting point for the introduction of the kinetic constraints, as shown in the following paragraph.

### MCR-ALS with kinetic constraints

The MCR-ALS method can be extended by incorporating kinetic equations as additional constraints (see SI 5). This approach offers two main advantages, *i.e.* (i) it provides optimized rate constants that best fit the experimental data and can be used to model the system, and (ii) it improves phase deconvolution by introducing additional constraints into the calculation. In this approach, the initial estimate corresponds to a concentration profile (*C*_0_) derived from a theoretical kinetic model, in contrast to the SIMPLISMA method, where initial profiles are calculated directly from experimental data. Still, SIMPLISMA results (Table 1) were used as exploratory analysis for the kinetic pathway.

**Fig. 4:**
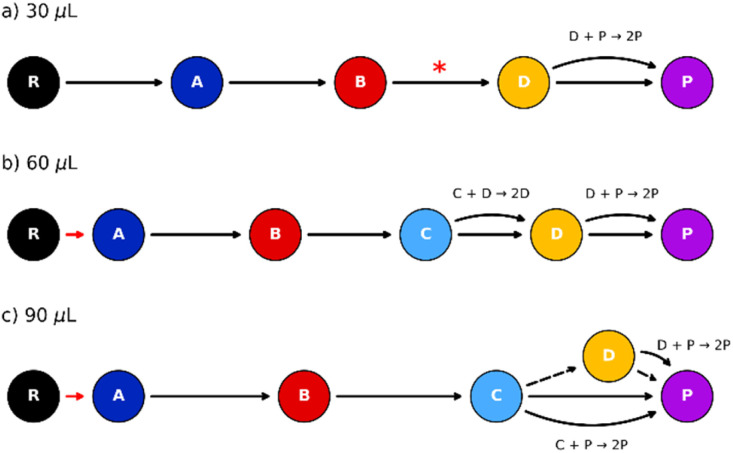
Reaction network depicting the pathway considered for the MCR-ALS with kinetic constraints of the 30 (a), 60 (b), 90 (c) µL experiment. Solid black arrow: main path; dashed black arrow: secondary path; curved arrow: autocatalytic step; red arrow: fast transition approximated as one component by the model; asterisk: time-delay included into the kinetic equation. Except for the merged step marked in red, all the transitions in black corresponded to a kinetic constant (*k*_i_) that was optimized by the calculation.

Starting with the apparently simpler 30 µL dataset, a more complex evolution, that did not conform to a straightforward R → A → B → D → P sequence, emerged (Fig. 4a). In particular, the last two components were not correctly modelled: both intermediate D and the product (P) appeared significantly delayed compared to the theoretical profiles, and this discrepancy was not attributable to a low kinetic constant. Intermediate D exhibited a rapid growth following an initial delayed (lag) phase, while the product displayed a sigmoidal increase. Finke and Watzky proposed a two-step kinetic model to rationalize sigmoidal kinetics, consisting of an initial nucleation step (D → P) followed by rapid autocatalytic growth (D + P → 2P). This model has been widely applied to transformations, including solid-state reactions.^44^ Incorporating the autocatalytic term improved the description of the product evolution and accounted for its sigmoidal behaviour. However, the rapid appearance of intermediate D required an additional treatment: we introduced a time-delay term in the corresponding differential equation, allowing the model to reproduce the observed lag phase before D begins to grow according to a traditional first order kinetics (Fig. SI9.1).

This delayed phase might be related to the induction periods that may occur during mechanochemical transformations, and can be explained considering the role played in the phase activation by the crystallite size-solvent interplay.^45^ Milling can reduce the particle size first before promoting the structural rearrangement, causing a delay in the phase's evolution observed by X-ray diffraction.^46,47^

With respect to the 60 µL system (Fig. 4b), the appearance of intermediate D was delayed as well, and it was properly addressed by including the autocatalytic term for both phases D and P, together with the addition of the new phase C into the main progression (Fig. SI9.2).

Finally, the evolution of intermediate D was even more unusual in the 90 µL dataset (Fig. 4c), as anticipated by the exploratory analysis. Despite its clear appearance following phase C, its limited growth ruled out a simple linear progression C → D → P. In this scenario, the incomplete formation of D could be modelled by assigning a low-rate constant to the C → D step, which, within the observed time frame, would results in the partial conversion of C and a final mixture of P and C (Fig. SI9.3). To align with experimental observation of pure Form II as the final product, both intermediates C and D must convert into P, making the formation of the CC_21 and CC_12 mixture a parallel secondary process.

**Fig. 5:**
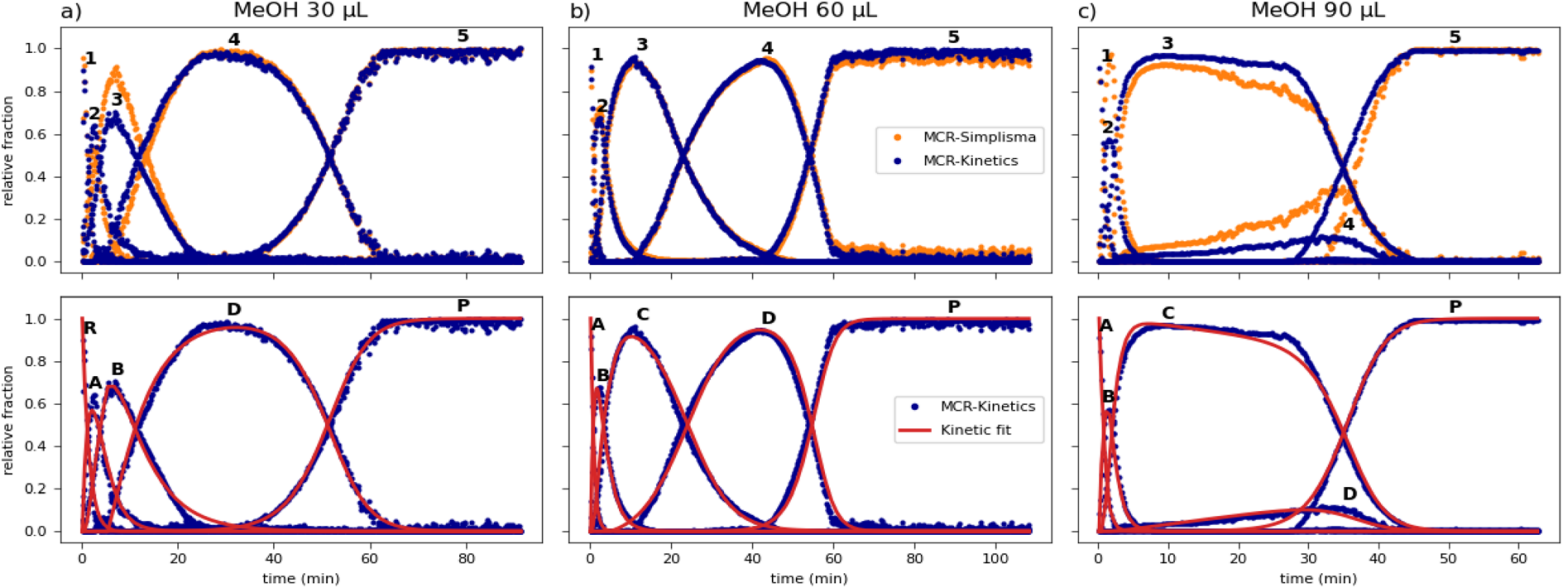
TOP: concentration profiles (*C* matrix) of the MCR-ALS SIMPLISMA model (orange) *vs.* MCR-ALS with kinetic constraint (blue) for the 30 (a), 60 (b), 90 (c) µL experiment; numbers identify each profile with the corresponding phase as in Table 1. BOTTOM: MCR-kinetic concentration profiles *vs.* kinetic fit optimized within the MCR-kinetic calculation for the 30 (a), 60 (b), 90 (c) µL experiment; letters identify each profile with the abbreviation used during the kinetic analysis as in Table 1.

Starting from the identified pathways, the full kinetic model was integrated as a hard constraint in the MCR-ALS algorithm (see SI 5.4). The top panels in Fig. 5 compare the MCR-kinetic results with the previous SIMPLISMA calculations for all three datasets. The strong agreement between the two methods confirms that the kinetic approach successfully reproduces the experimental data (Fig. SI10.1). Minor discrepancies remain for component three (B) in the 30 µL dataset and component four (D) in the 90 µL dataset. These differences correspond to an improvement in phase deconvolution (Fig. SI11), which makes the calculated concentration profiles more representative of the pure chemical phases, with the disappearance of CC-1 : 2 peaks for components three, four, and five (Fig. 5c, top). This implementation of MCR-ALS also returned optimized rate constants of the kinetic models employed for the resolution (see SI 10). The bottom panels of Fig. 5 compare the concentration profiles derived from MCR-ALS (blue) with the theoretical profiles (red) calculated from the optimized rate constants. Given the chemical validity of the MCR calculation ensured by good deconvolution, the excellent match between the deconvolution profiles and the optimized theoretical model provides a twofold confirmation: it validates the proposed kinetic models and reinforces the reliability of the overall deconvolution, as the profiles fit consistently within a mechanistic framework.

While the MCR-derived kinetic models successfully describe the system and its phases evolution, a further step was considered to relate the insights here derived to the mechanistic aspects of mechanochemical processing, with a special focus on the role of the solvent in the mechanical activation and kinetic process.

### Kinetic modelling

The kinetic curves can be further rationalized by applying a kinetic model based on a statistical description of the mechanical processing of powders in ball mills. The starting assumption of the model is that the reactants transform into the product when the powders experience critical loading conditions (CLCs) in irregularly distributed small volumes, v*(see SI 6).^48–50^

The best-fit analysis of the kinetic data provides insight into the role of LAG (Liquid Assisted Grinding) on both mechanical activation and the resulting chemistry (see SI 12).

In terms of mechanical activation, the fitting procedure allows estimation of the fraction of powder experiencing CLCs per impact, *k*, which is found to be independent from the amount of LAG added. This indicates that LAG does not alter the fraction of material effectively processed by the mechanical treatment. In all cases, this fraction lies in the narrow range of 1.6–1.9% of the total powder mass inside the reactor.

**Fig. 6:**
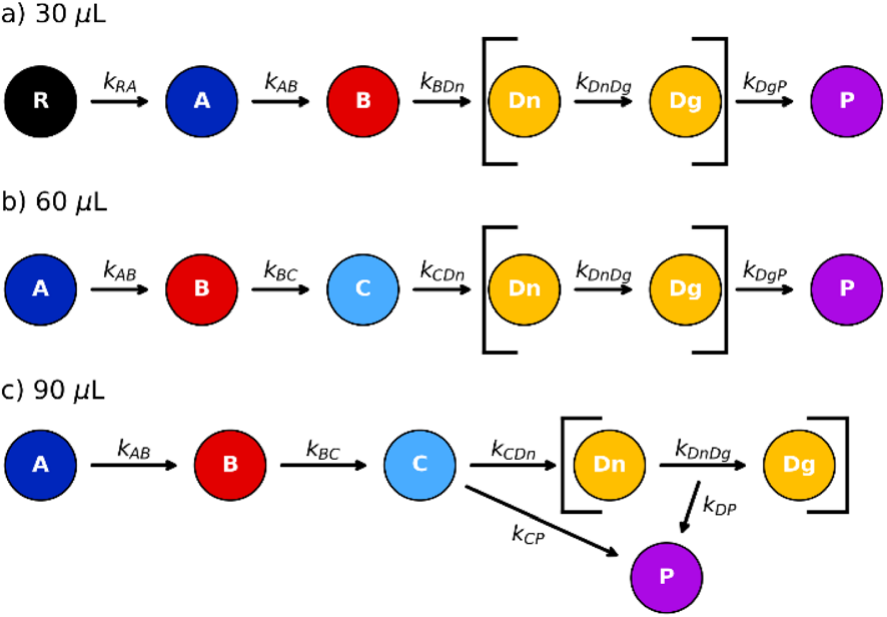
Scheme of the kinetic equations derived from the kinetic modelling (details in SI).

In contrast, clear differences emerge in the reaction pathways. The local kinetic schemes required to describe the data differ among the three cases, indicating that the amount of LAG modifies the reaction mechanism rather than the mechanical activation step. This is reflected in the selective formation of intermediates and in changes in the relative rates of individual transformations. For instance, at the lowest LAG content, the initial transformation from R to A is kinetically resolved, indicating that this early step proceeds at a measurable rate (Fig. 6a). At higher LAG amounts, this transformation becomes too fast to be detected, and R is no longer observable at the beginning of the measurement. At intermediate and high LAG contents, *i.e.* 60 µL and 90 µL, C is formed as an intermediate, whereas it is not detected at lower LAG levels. The dependence of C on the amount of LAG is evident not only from its formation, but also from its subsequent fate. At the final stage of the reaction, the transformation of C strongly depends on the LAG content. When 60 µL of LAG is used, C is fully converted into D, which subsequently yields the final product P (Fig. 6b), whereas at the highest LAG content 90 µL, C is partially converted into D and partially directly into P (Fig. 6c).

Despite these mechanistic differences, several reaction steps are common to all systems and enable a meaningful comparison. In particular, the transformations A → B, D_*n*_ → D_*g*_, and D → P are consistently observed across all the datasets. This allows direct comparison of the corresponding rate constants *k*_AB_, *k*_D_*n*_D_*g*__, and *k*_DP_, as well as of the fraction *α*_P,start_ formed during the very first impact of the mechanochemical reactions.

**Fig. 7:**
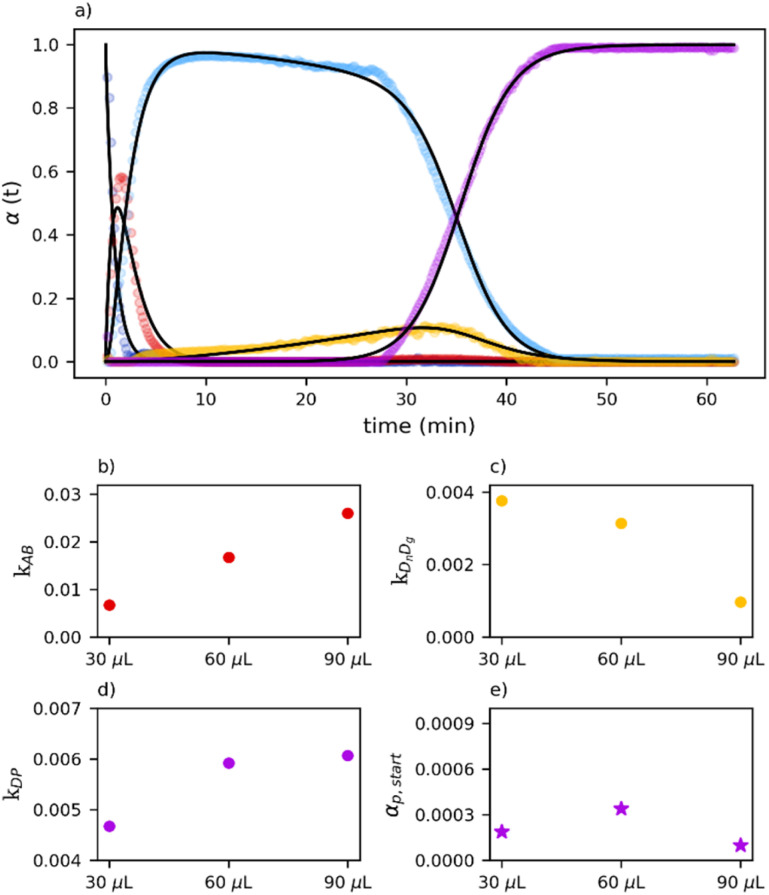
On top, best fit for the 90 µL dataset to show the ability of the model to captures the data (a). At the bottom, comparison of the fitted kinetic parameters as a function of the amount of LAG added. Apparent rate constants for (b) the A → B reaction step, *k*_AB_, (c) the D → P reaction step *k*_DP_, and (d) the D_*n*_ → D_*g*_ reaction step, are reported for 30, 60, and 90 µL of LAG. Panel (e) shows the fraction *α*_P,start_ of phase P formed during the very first effective impact.

As highlighted in Fig. 7, a nearly linear increase of the apparent single-impact rate constant for the A → B transformation with increasing solvent content is observed. Notably, the apparent kinetics reveal an inverse relationship between the rate constants associated with the catalytic growth of D and the catalytic formation of P. The amount of form *α*_P,start_ is almost constant for all the different datasets.

Overall, these trends indicate that interactions between the solid phases and the liquid additive play a significant role within the mechanochemical framework. At the same time, the persistence of common reaction steps across all conditions suggests that LAG reshapes the reaction network by promoting or suppressing specific pathways, while preserving a core set of mechanochemical transformations.

## Conclusions

This work addressed a central challenge in scaling up mechanochemical syntheses, namely the derivation of reaction kinetics when unpredictable formation and complex interplay of multiple crystalline phases occur during solid-state transformations.

Using the co-crystallisation of CSBA and INA as a model system, we showed step by step how to build up a robust workflow aimed to scale up mechanochemical reactions.

At first *via* PXRD *in situ* monitoring we could (i) detect the complex interplay between the several crystal structures occurring, (ii) identify and quantify a new, unknown compound, and (iii) have evidence of the LAG role in the reaction profile.

The MCR-ALS method then helped in detecting carefully the phase transformations occurring during the milling and served as an exploratory analysis for the kinetic pathway.

By integrating kinetic modelling as soft-hard constraint in the MCR-ALS workflow, we then improved the deconvolution and quantification of overlapping phases, we quantitatively resolved the evolution of crystalline intermediates under varying methanol-assisted conditions, and we could rationalize the derived kinetics.

Finally, the established MCR-ALS workflow was validated by applying a phenomenological kinetic modelling tailored to rationalize the mechanochemical reaction rates.

Notably, the methodology can be applied to several kind of data, crystalline or amorphous materials, thus expanding the suitability of this workflow to different fields. In fact, being the MCR-ALS method widely applied to spectroscopic techniques, the same approach could, in principle, be extended to monitor amorphization processes using methods such as Raman spectroscopy, which have already been employed in the monitoring of mechanochemical studies. To the best of our knowledge, however, such approaches has not yet been used to extract kinetic information.

In conclusion, we believe that we proposed a robust workflow for the scale up of the mechanochemical reactions, and we hope that this study will help facilitating the translation of mechanochemical methodologies from laboratory to industrial applications.

## Author contributions

LM, FE and LC conceived the project; LC performed the experiments; LM, MC and FD analyzed data; LC and FE supervised the project. All authors discussed the results, wrote and commented on the manuscript.

## Conflicts of interest

There are no conflicts to declare.

## Supplementary Material

SC-017-D6SC01158F-s001

## Data Availability

The experimental data for this article, including the raw synchrotron data, are available in Zenodo at 10.5281/zenodo.18596362. Supplementary information (SI): materials and methods section, and information about extraction and modelling of kinetic data. See DOI: https://doi.org/10.1039/d6sc01158f.
