# Supplementary material for: Scaling up mechanochemical reactions: linking crystalline phase evolution studied via in situ PXRD with kinetics from MCR-ALS
Source: Chem Sci. 2026 May 12;17(24):12047–54. doi: 10.1039/d6sc01158f (PMC13164414; doi:10.1039/d6sc01158f)
Supplement: SC-017-D6SC01158F-s001 [file SC-017-D6SC01158F-s001.pdf]

Supplementary Information (SI) for Chemical Science.
This journal is © The Royal Society of Chemistry 2026

# Electronic Supplementary Information

## Scaling Up Mechanochemical Reactions: Linking Crystalline Phase Evolution Studied via in Situ PXRD with Kinetics from MCR-ALS

Laura Macchietti,*[a] Mara Carta,[b,c] Francesco Delogu,[b,c] Fabrizia Grepioni,[a] Franziska Emmerling[d,e] and Lucia Casali*[d]

[a] Department of Chemistry `Giacomo Ciamician', University of Bologna, Via Gobetti 85, 40129 Bologna, Italy.

[b] Department of Mechanical, Chemical and Materials Engineering, University of Cagliari, via Marengo 2, 09123 Cagliari, Italy.

[c] Center for Colloid and Surface Science (CSGI), Cagliari Research unit, Department of Chemistry, University of Florence, via della Lastruccia 3, 50019 – Sesto Fiorentino, FI, Italy.

[d] Federal Institute for Materials Research and Testing, Richard-Willstätter-Straße 11, 12489 Berlin, Germany.

[e] Department of Chemistry, Humboldt-Universität zu Berlin, 12489 Berlin, Germany

## Table of Contents

**Materials and Methods**

1. Reagents 1
2. Mechanochemical Synthesis 1
3. In-situ PXRD 1

**Data Analysis**

4. MCR-ALS method 2
5. Analysis Workflow 2
5.1 Data pre-processing 2
5.2 MCR-ALS Soft Modelling 2
5.3 Kinetic Model Identification 3
5.4 MCR-ALS with Kinetic Constraints (Hard-Soft Modelling) 3
6. Computational Details 3
7. Kinetic modelling 4

**Experimental Data**

8. Comparison of MCR-ALS-SIMPLISMA profiles with reference data 6
9. Kinetic model identification 12
10. MCR-ALS model evaluation 14
11. Comparison between MCR-ALS and MCR-ALS with constraints (C4 at 90 μL) 16
12. Kinetic Fitting 17

**References**

**Materials and Methods**

**1. Reagents**

Chloro-3-sulfamoylbenzoic acid (CSBA) and Isonicotinamide (INA) were purchaised from BLDPharma and TCI, respectively, and used without further purification.

Crystallographic information about the binary systems forming during the mechanochemical reactions between CSBA and INA were found in CSD database: 1CSBA-1INA co-crystal Form I (refcode DIPWIM), 1CSBA-1INA co-crystal Form II (refcode DIPWIM01), 1CSBA-2INA co-crystal (refcode DIPMUY), 2CSBA-1INA co-crystal (refcode DIPWEI). Hereafter we mainly refer to these compounds as following: FI, FII, CC_12, CC_21.

**2. Mechanochemical synthesis**

CSBA (263.4 mg) and INA (136.6 mg) were placed in a 14 mL perfluoroalkoxy polymer (PFA) jar containing a stainless-steel ball with a diameter of 9 mm. 30 µL to 90 µL of MeOH were added with a micropipette, and the samples were then milled at 25 Hz for 100 min.

**3. In situ XRD**

In situ XRD measurements were conducted using an X-ray energy of 60 keV (λ = 0.207 A˚) at the Powder Diffraction and Total Scattering Beamline P02.1 at the Deutsches Elektronen-Synchrotron (DESY), employing a modified IST-636 mixer mill (InSolido Technologies, Croatia, Zagreb) operating at a frequency of 25 Hz. The X-ray beam was set to pass through the bottom of the PFA reaction vessel. Exposure time was set to 20s. Diffraction data were collected on a PerkinElmer XRD1621 flat-panel detector positioned 1595 mm from the sample, which consisted of an amorphous Si sensor equipped with a CsI scintillator (pixel number: 2048 × 2048, pixel size: 200 × 200 m2). To obtain the classic one-dimensional powder XRD pattern, the two-dimensional diffraction images were using a Python script developed in-house. Two-dimensional time-resolved of in situ monitoring data were created in Python and the background of each diffraction pattern was subtracted prior to plotting.

**Data analysis**

**4. MCR-ALS method**

The multivariate curve resolution (MCR) algorithm decomposes the data matrix $D_{(sxn)}$ (*n* variables, *s* observations) into the k-components matrix $S^T_{(kxn)}$ and the respective concentration profiles $C_{(sxk)}$, according to the bilinear model:

$$D_{(sxn)} = C_{(sxk)} \text{ X } S^T_{(kxn)} + E_{(sxn)}$$

where E represents the residuals matrix accounting of the difference between the model and the original data.

This resolution is inherently affected by a degree of uncertainty due to the existence of different sets of concentrations and signals profiles that can satisfy the proposed equation with equal goodness of fit. Different types of ambiguities are identified according to the source of the uncertainty. The most relevant are the rotational ambiguity, referring to the possibility of different shapes for the C and $S^T$ matrix and therefore affecting their physical interpretation, and the intensity ambiguity, concerning the relative scales between the two matrices with consequences on the quantitative interpretability.

As the intent of MCR is to provide a chemically meaningful results, the resolution ambiguities are reduced by including mathematical constraints to guide the matrix computation towards a physically meaningful outcome.

The MCR-ALS method performs the optimization and inclusion of constraints through the iterative implementation of alternating least squares (ALS). Moreover, this implementation requires an initial estimate of either the concentration profiles ($C_0$) or the pure component profiles ($S_0$) to start the computation. The initial estimate is key in reducing the rotational ambiguity and should be sufficiently representative of the system; different algorithms are available to derive suitable initial estimates directly from the data variability without detailed knowledge of the profiles shapes. The conditions used in the present work are reported in the following section, whereas for further information on the subject the reader is referred to dedicated works.[1,2]

In addition to the traditional MCR soft-modelling approach, consisting of data-driven analysis with minimal assumptions about the system, constraints enable a combined soft-hard modelling strategy suitable for kinetic studies.[3] This approach refines concentration profiles and allows reliable estimation of rate constants by incorporating explicit kinetic equations (hard modelling) into the ALS optimization.

Due to the rotational ambiguity and the founding soft-modelling approach to the resolution, the intrinsic variability of the D matrix critically influences the deconvolution performance of MCR-ALS through its association to rank deficiency. Rank deficiency arises when the number of linearly independent contributions in the data is lower than the actual number of chemical species, typically due to overlapping concentration or spectral profiles.[4] Although several approaches can mitigate this issue,[5] some conditions inherently produce rank-deficient data, i.e. in solid-state transformations forming a co-crystal, the reactants evolve with identical concentration profiles and are therefore indistinguishable to bilinear models.

Fast kinetic events[6] and insufficient sampling density[7] have also been reported to reduce the effective variability in the data. When a transformation occurs too quickly relative to the acquisition rate, the species' evolution is poorly represented, and the variability needed to

resolve separate concentration profiles becomes insufficient, ultimately contributing to rank-deficiency-related resolution failure.

## 5. Analysis Workflow

### 5.1 Data Pre-processing

Prior to chemometric analysis, the background corrected dataset was area normalized, and the region between 1.24 and 1.32 nm, containing the jar contribution, was excluded.

### 5.2 MCR-ALS Soft-Modelling

An exploratory analysis of the experimental data was conducted to confirm phase transitions and identify key kinetic features. Initial estimates of pure profiles ($S_0$) were obtained using the SIMPLISMA algorithm.[8] This step includes the selection of the number of components considered for the resolution. Given the initial information about the datasets, models were computed starting from four and up to six components. Non-negativity constraints were applied to both concentration and pure profiles, and a closure constraint was imposed on concentrations (i.e., maintaining the total sum across components) to suppress the intensity ambiguity. The best model was selected evaluating the chemical relevance of the resolution: pure profiles ($S^T$) were validated by comparison with reference X-ray diffraction patterns calculated from single-crystal data, ensuing the correspondence between the calculated components and chemically pure phases.

### 5.3 Kinetic Model Identification

Based on the observed phase progression and its agreement with experimental data, a kinetic model was proposed and expressed as a system of differential equations. For example, the sequence $A \xrightarrow{k_1} B \xrightarrow{k_2} C$ corresponds to:

$$\frac{dA}{dt} = -k_1 A\,,\quad \frac{dB}{dt} = k_1 A - k_2 B,\quad \frac{dC}{dt} = k_2 B$$

An initial estimate of the rate constants was identified to reproduce the concentration profiles obtained from the soft-modelling step and reflecting the system’s kinetics.

### 5.4 MCR-ALS with Kinetic Constraints (Hard–Soft Modelling)

The identified kinetic model was incorporated as a hard constraint in the MCR-ALS optimization, providing the initial concentration estimate ($C_0$). Non-negativity constraints were again applied to both concentration and pure profiles.
The lack of fit (LOF) was used as the primary indicator of resolution quality, calculated for both soft and hard–soft models as:

$$LOF\,(\%) = 100 \cdot \sqrt{\frac{\sum_{ij} e_{ij}^2}{\sum_{ij} x_{ij}^2}},\; e_{ij} = x_{ij} - \hat{x}_{ij}$$

where $x_{ij}$ is an element of the original data matrix, $\hat{x}_{ij}$ the corresponding value reconstructed from the model (C X ST = $\hat{X}$), and $e_{ij}$ the residual. Pure profiles (ST), concentration profiles (C), and the optimized kinetic model were compared against soft-modelling results and experimental data to confirm chemical validity. If discrepancies were observed, the kinetic model was refined until satisfactory agreement was achieved.

## 6. Computational Details

Data pre-processing and chemometric computations were performed in Python (v3.11.7) using the Spectrochempy library as the core framework for MCR-ALS analysis. Kinetic constraints were implemented following the hard–soft modelling strategy described in the library documentation,[9] with modifications to accommodate time-delayed kinetic profiles. Custom functions were integrated into the MCR-ALS optimization, and time-delayed kinetics were solved using the ddeint package for delay differential equations.

## 7. Kinetic modelling

Analysis of the experimental kinetic data was performed using a phenomenological analytical model based on the statistical character of the mechanical processing by ball milling. During milling, impacts between the milling tools stochastically trap small volumes of powder between the colliding surfaces, generating mechanical stresses at interparticle contacts within the trapped powder volume. As stress intensity exceeds the yield stress, plastic deformation takes place. Critical loading conditions (CLCs) can be assumed to occur in irregularly distributed small volumes $v^*$ · It is within volumes $v^*$ that the reactant transforms into the product. The involvement of powders in impacts is stochastic.

Therefore, volumes $v^*$ are also affected by CLCs stochastically. It follows that volumes $v^*$ that have been affected by CLCs a different number of times coexist within the total volume $V$ of powder inside the reactor. Volumes $v^*$ involved in a different number of CLCs can be expected to exhibit a different product molar fraction, $\alpha_i$. Then, the global kinetics of the mechanically activated transformation can be satisfactorily rationalized only once local kinetics is combined with a suitable statistical framework. To this aim we assume that:

1. the vigorous stirring of the powders keeps the powder charge homogeneous throughout the milling process;
2. at each impact, only a small volume $v$ of the total powder inside the reactor, $V$, is subjected to critical loading conditions (CLCs) able to induce the transformation;
3. the fraction of powder affected by CLCs, $\kappa = \frac{\sum v^*}{V}$ remains constant throughout the entire process;
4. the local transformation degree, $\alpha_i$, in volume $v$ changes with the number $i$ of CLCs the volume experiences.

The volume fraction of powder affected by CLCs $i$ times after $m$ impacts, $\chi_i(m)$, can be expressed as

$$\chi_i(m) = \left[\frac{(\kappa m)^i}{i!}\right]\exp(-\kappa m) \qquad (1)$$

The global transformation degree, $\alpha(m)$, is equal to the sum of the local transformation degrees, $\alpha_i$, weighted by the volume fractions $\chi_i(m)$ of powder that have experienced CLCs $i$ times. Accordingly,

$$\alpha(m) = \sum_{i=1}^{\infty} \chi_i(m)\alpha_i \qquad (2)$$

Considering that the total number of impacts, $m$, can be simply expressed as the product $f\,t$ between impact frequency, $f$, and time, $t$, Eq. 1 can be re-written in the time-dependent form

$$\chi_i(t) = \left[(\kappa f t)^i/i!\right] exp(-\kappa f t) = \left[(k t)^i/i!\right] exp(-k t) \qquad (3)$$

Where $k$ is the volume fraction of powder affected by CLCs per unit time. Eq. 2 can be also re-written as

$$\alpha(t) = \sum_{i=1}^{\infty} \chi_i(t)\alpha_i \qquad (4)$$

The way the local transformation degree, $\alpha_i$, changes with the number $i$ of CLCs depends on the chemical nature of the system.

**Experimental data**

## 8. Comparison of MCR-ALS-SIMPLISMA profiles with reference data

**Fig.ESI8.1.** Comparison between the pure profiles ($S^T$ matrix) obtained from the MCR-ALS calculation on the 30 µL data and the reference patterns (coloured lines). The dotted line indicates a region of limited relevance, dominated by the jar contribution.

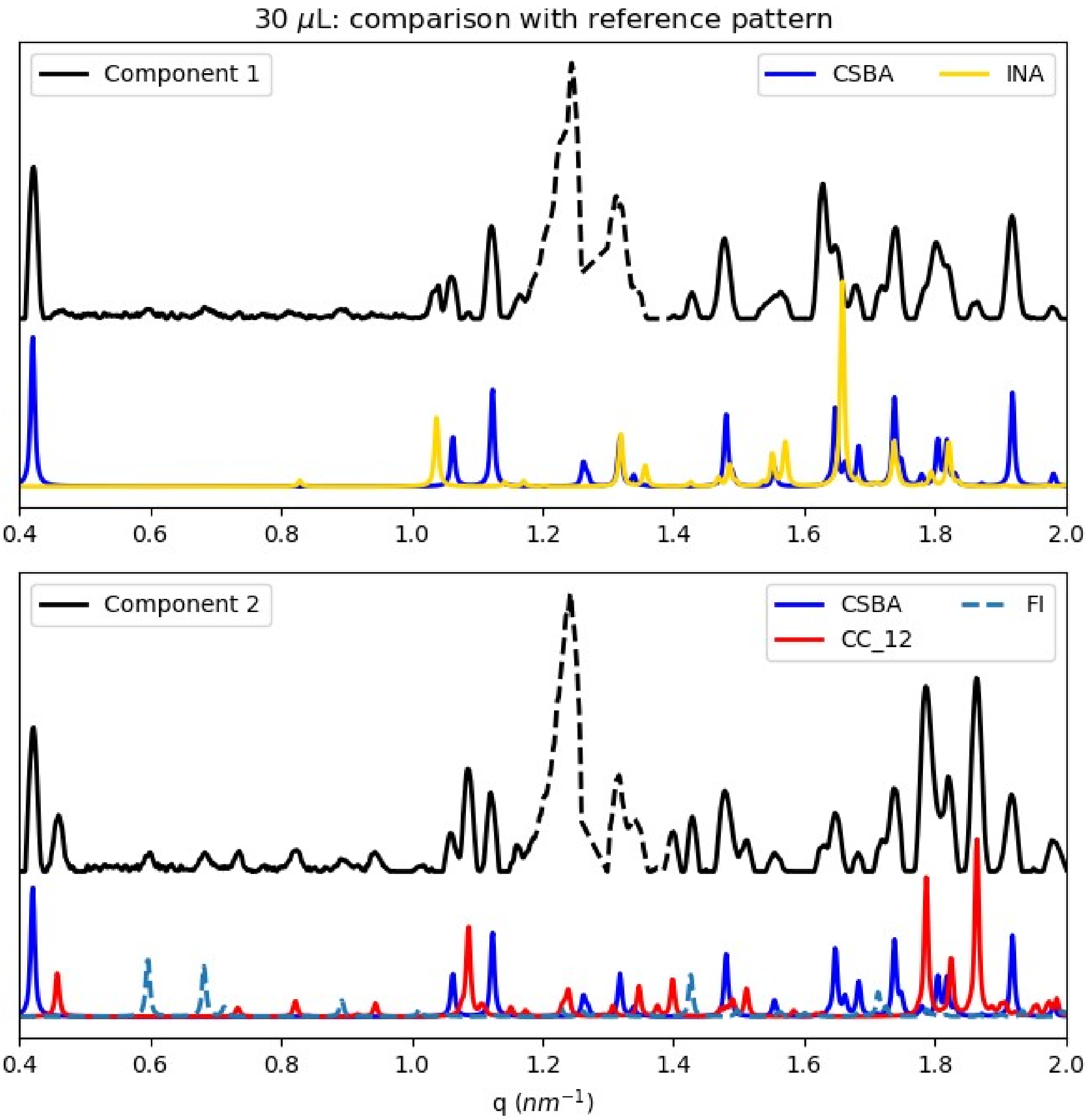
30 $\mu$L: comparison with reference pattern
Component 1
CSBA
INA
Component 2
CSBA
FI
CC_12
0.4
0.6
0.8
1.0
1.2
1.4
1.6
1.8
2.0
q ($nm^{-1}$)

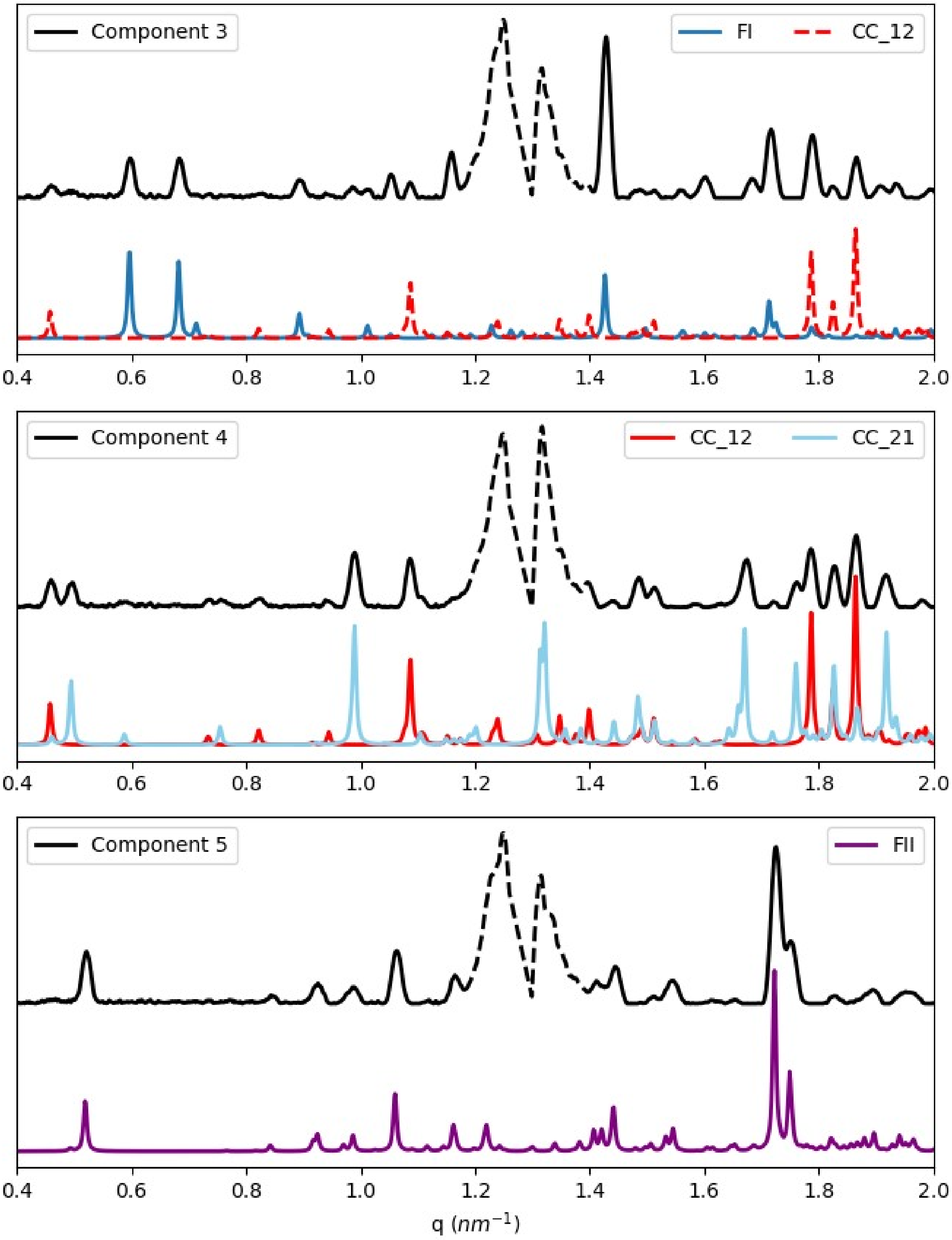
Component 3
FI
CC_12
Component 4
CC_12
CC_21
Component 5
FII
0.4
0.6
0.8
1.0
1.2
1.4
1.6
1.8
2.0
q ($nm^{-1}$)

**Fig.ESI8.2.** Comparison between the pure profiles ($S^T$ matrix) obtained from the MCR-ALS calculation on the 60 µL data and the reference patterns (coloured lines). The dotted line indicates a region of limited relevance, dominated by the jar contribution.

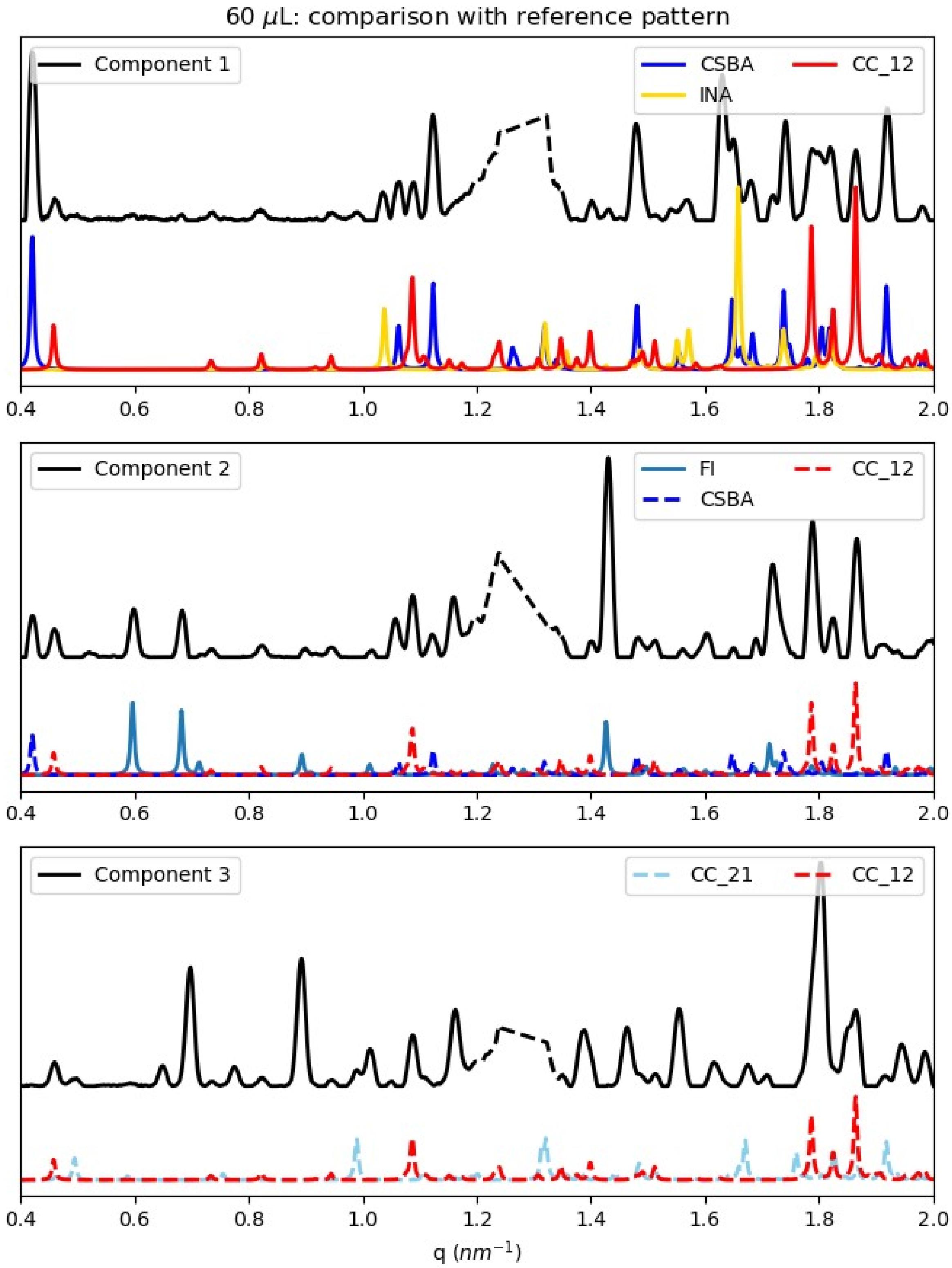

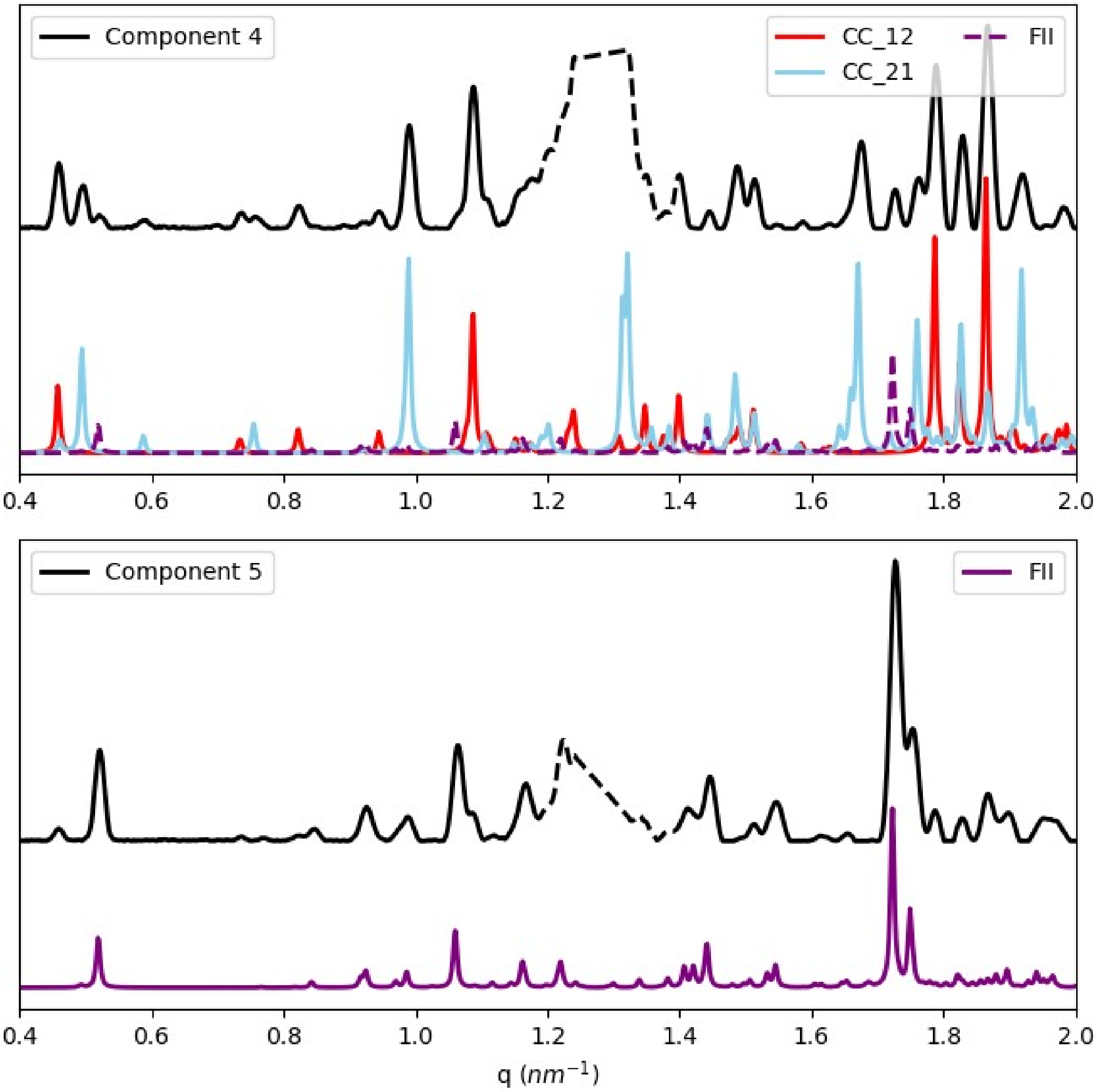
Component 4
CC_12
CC_21
FII
0.4
0.6
0.8
1.0
1.2
1.4
1.6
1.8
2.0
Component 5
FII
0.4
0.6
0.8
1.0
1.2
1.4
1.6
1.8
2.0
q ($nm^{-1}$)

**Fig.ESI8.3.** Comparison between the pure profiles ($S^T$ matrix) obtained from the MCR-ALS calculation on the 90 μL data and the reference patterns (coloured lines). The dotted line indicates a region of limited relevance, dominated by the jar contribution.

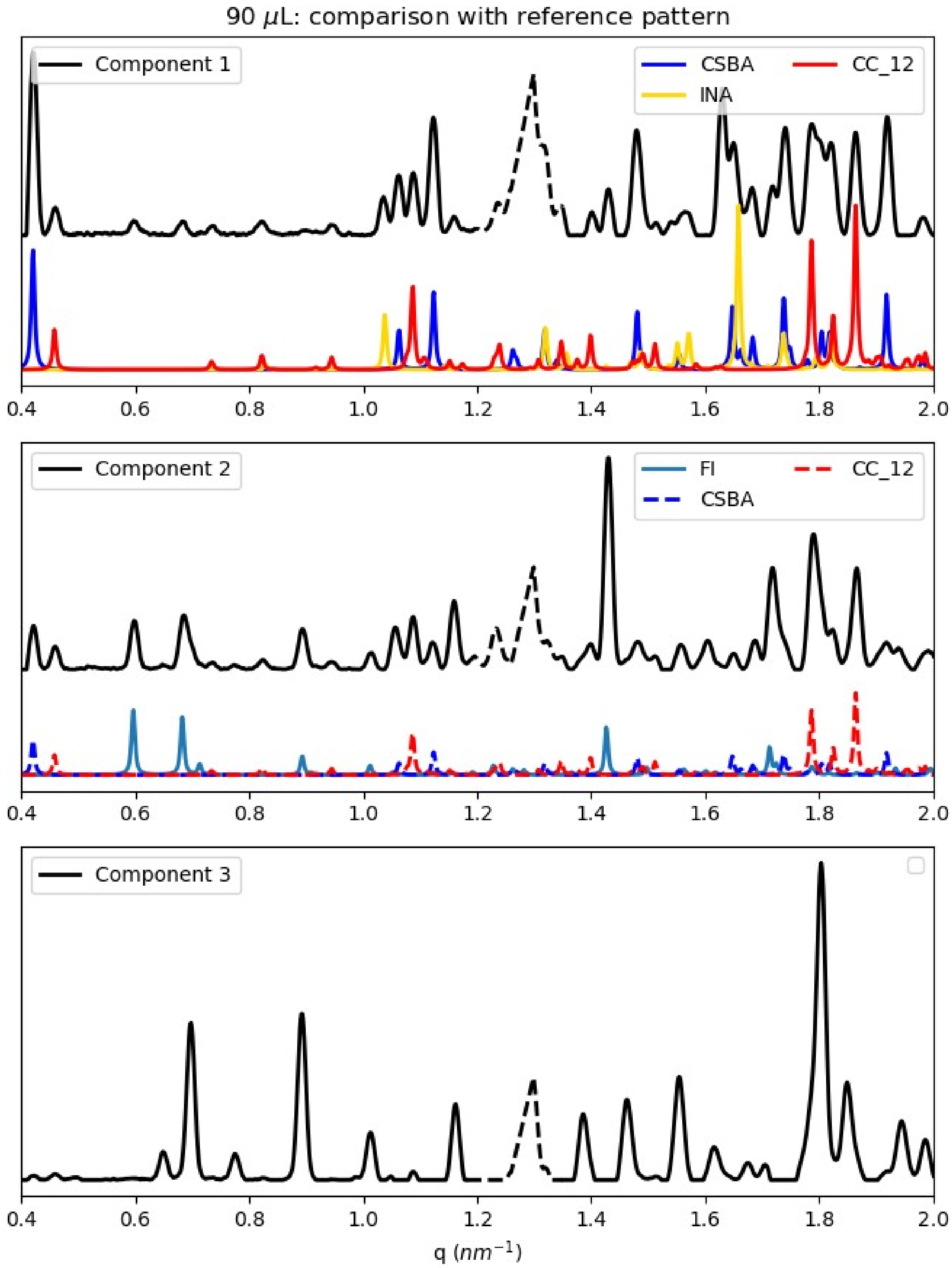

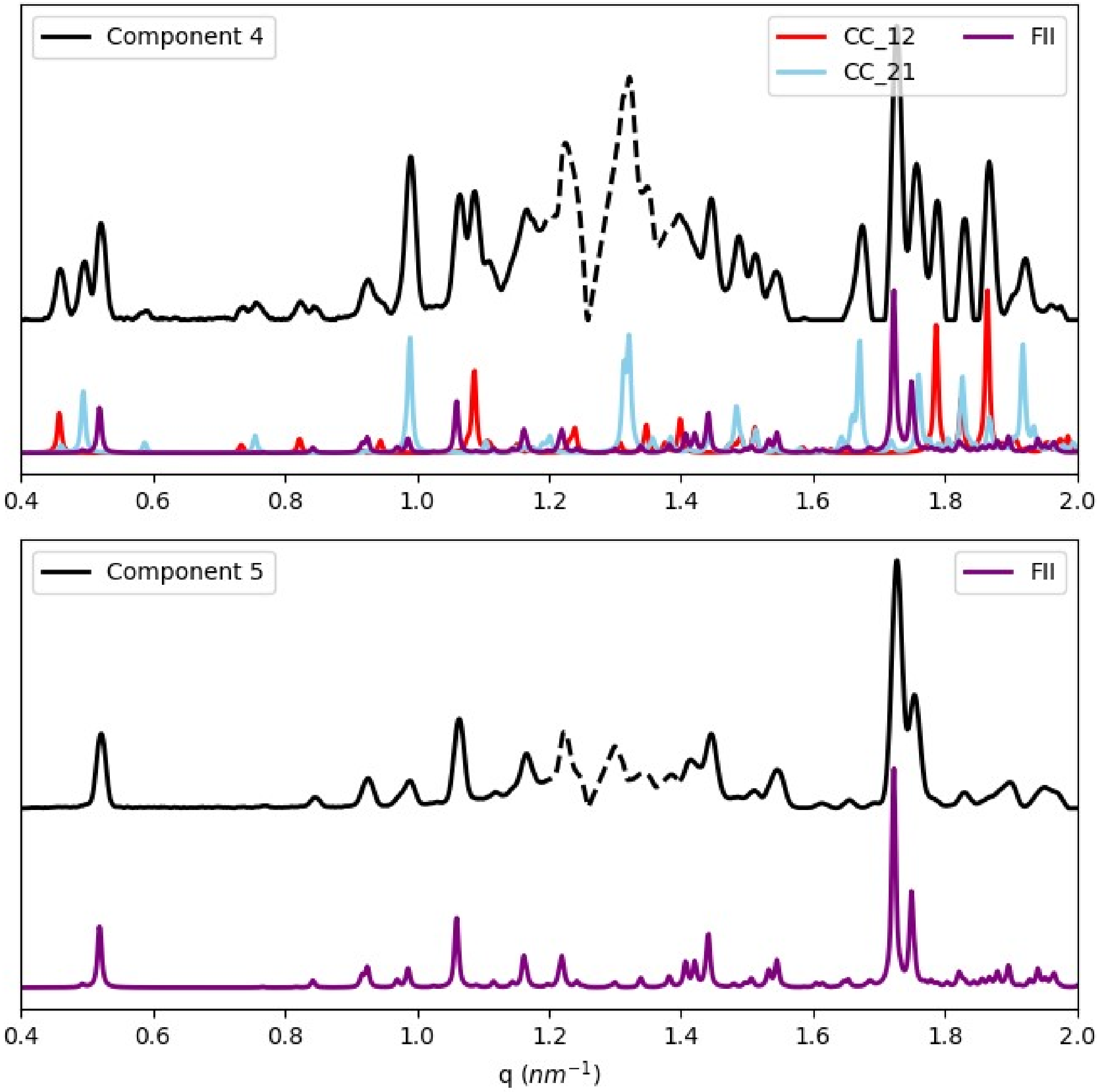
Component 4
CC_12
CC_21
FII
0.4
0.6
0.8
1.0
1.2
1.4
1.6
1.8
2.0
Component 5
FII
0.4
0.6
0.8
1.0
1.2
1.4
1.6
1.8
2.0
q ($nm^{-1}$)

## 9. Kinetic model identification

**Fig.ESI9.1.** Comparison of the MCR-ALS (SIMPLISMA) concentration profiles (black) from the 30 µL dataset with theoretical profiles (coloured lines) from the kinetic model: (a) R → A, A → B , B → D, D → P ; (b) R → A, A → B , B →* D, D → P, D+P →2P. * = time-delay

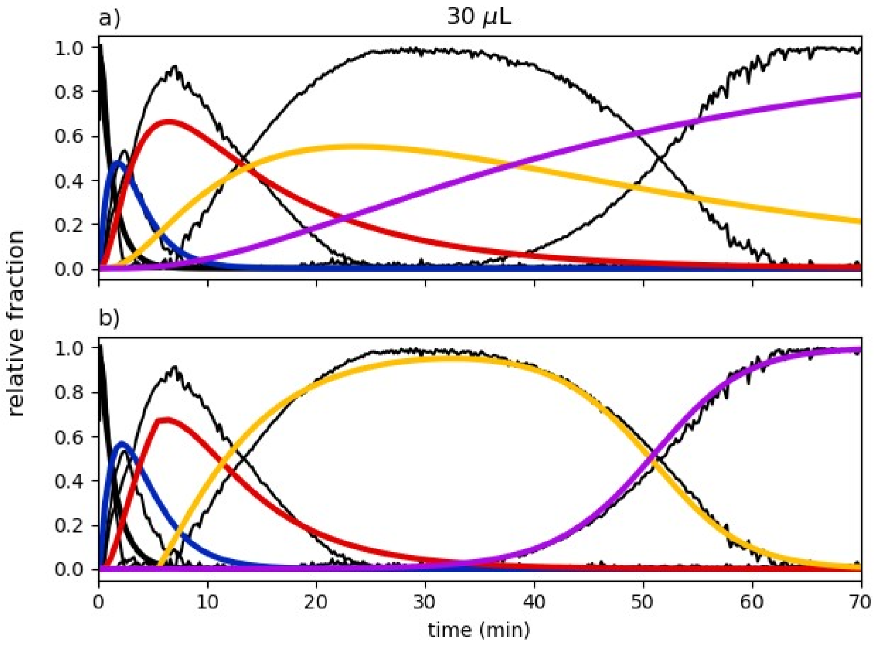


**Fig.ESI9.2.** Comparison of the MCR-ALS (SIMPLISMA) concentration profiles (black) from the 60 µL dataset with theoretical profiles (coloured lines) from the kinetic model: (a) A → B , B → C,C → *D, D → P, D+P →2P ; (b) A → B , B → C, C →D, C +D →2D. D → P, D+P →2P. * = time-delay

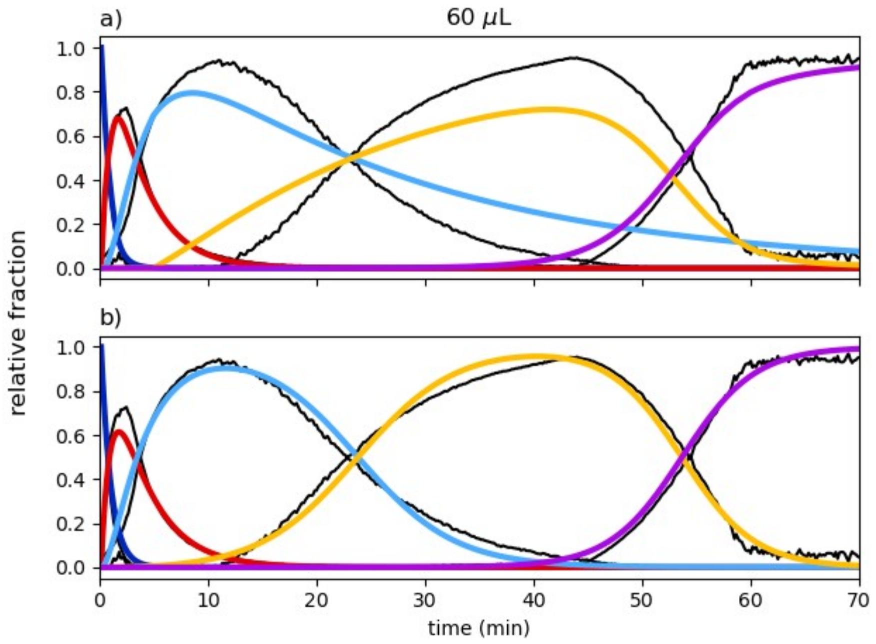

**Fig.ESI9.3.** Comparison of the MCR-ALS (SIMPLISMA) concentration profiles (black) from the 90 µL dataset with theoretical profiles (coloured lines) from the kinetic model: (a) A → B , B → C, C →D, D → P, D+P →2P ; (b) A → B , B → C, C →D, C →P. D → P, C+P →2P, D+P →2P.

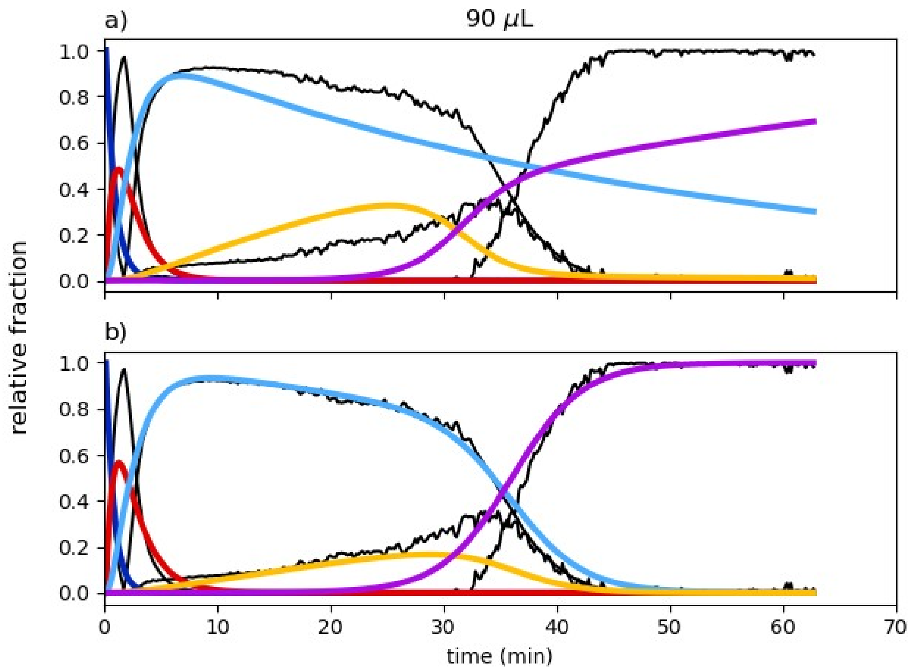

## 10. MCR-ALS model evaluation

**Table ESI10.1.** Optimized rate constants.

| **DATASET 30 ML** | **B>D** | **R>A** | **A>B** | **B>D** | **D>P** | **D+P>2P** |
|---|---|---|---|---|---|---|
| | delay | **k1** | **k2** | **k3** | **k4** | **k5** |
| | 5.5 | 0.615 | 0.300 | 0.135 | 0.0001 | 0.230 |

| **DATASET 60 ML** | **A>B** | **B>C** | **C>D** | **C+D>2D** | **D>P** | **D+P>2P** |
|---|---|---|---|---|---|---|
| | **k1** | **k2** | **k3** | **k4** | **k5** | **k6** |
| | 1 | 0.298 | 0.0056 | 0.187 | 2.92E-05 | 0.325 |

| **DATASET 90 ML** | **A>B** | **B>C** | **C>D** | **C>P** | **D>P** | **C+P>2P** | **D+P>2P** |
|---|---|---|---|---|---|---|---|
| | **k1** | **k2** | **k3** | **k4** | **k5** | **k6** | **k7** |
| | 1.05 | 0.700 | 0.0045 | 7.20E-06 | 0.000501 | 0.327 | 0.350 |

**Table ESI10.2**. Lack of fit obtained for the MCR-ALS models.

| | Lack of Fit (LOF) | |
|---|---|---|
| **Dataset** | **MCR-SIMPLISMA** | **MCR-Kinetics** |
| **30 µL** | 3.3 | 3.6 |
| **60 µL** | 3.2 | 3.2 |
| **90 µL** | 4.8 | 5.1 |

**Fig.ESI.10.1.** Plot of merit for the MCR-ALS calculation with kinetic constraints.

a) 30 µL dataset

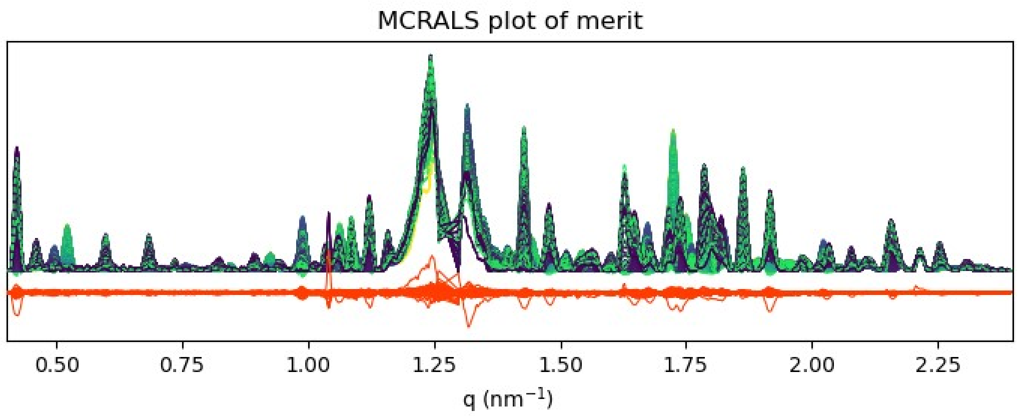


b) 60 μL dataset

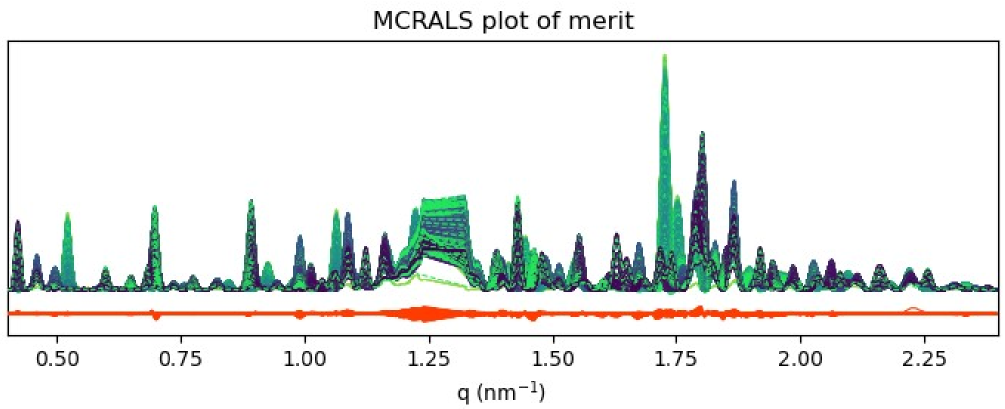


c) 90μL dataset

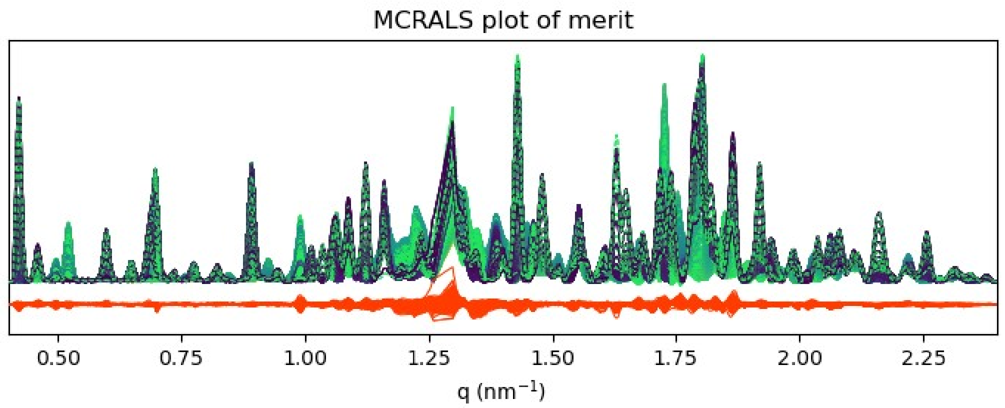

## 11. Comparison between MCR-ALS and MCR-ALS with constraints (C4 at 90 µL)

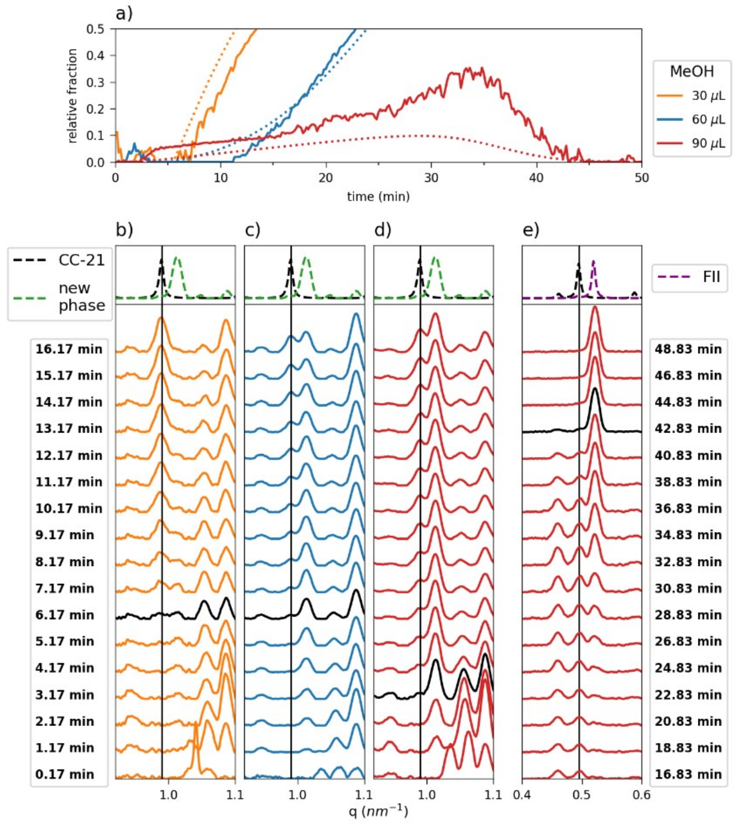


**Fig.ESI.11.1.** a) MCR-ALS (SIMPLISMA) concentration profiles of component C4 (solid line) and corresponding theoretical profiles optimized with MCR kinetic constrain (dotted line). b-e) Experimental in-situ data for the 30 µL (b), 60 µL (c) and 90 µL (d-e) test, registered between 0 and 16 min of the grinding (b-d) and between 16 and 48 min (e), showing the appearance (b-d) and disappearance (e) of the 2:1 co-crystal, key time point marked as black. Calculated patterns from single crystal structures (CC-2:1 and form II) and MCR-C3 profile (new phase) are reported as a reference (dotted lines).

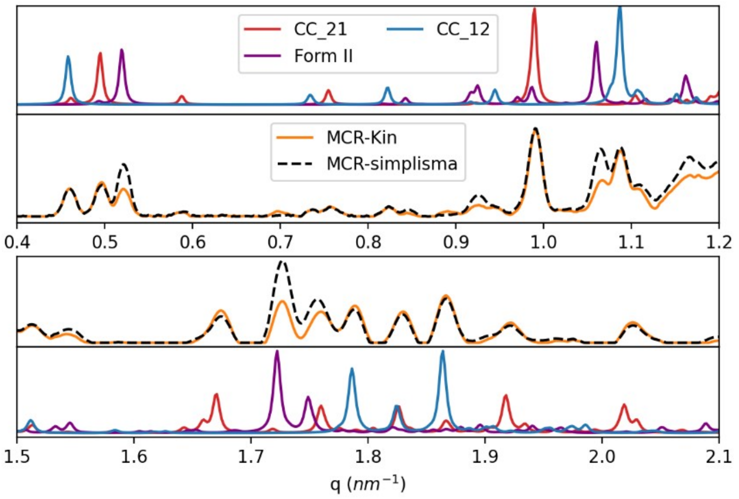


**Fig.ESI.11.2.** Comparison of component C4 pure profile of the MCR-ALS SIMPLISMA calculation (black dotted line) and MCR-ALS with kinetic constraint (orange line) from the 90 µL dataset. Calculated patterns (red, blue, and purple lines) are shown as reference.

## 12. Kinetic Fitting

**30 µL experiment.** The simplest kinetic scheme able to account for the experimental curves involves parallel and consecutive reactions as shown below:

$$R \xrightarrow{k_{RA}} A \xrightarrow{k_{AB}} B \xrightarrow{k_{BD}} \left[D_n \xrightarrow{k_{D_nD_g}} D_g\right] \xrightarrow{k_{DP}} P$$

Here, $k_{RA}$, $k_{AB}$, $k_{BD}$, $k_{D_nD_g}$ and $k_{DP}$represent apparent rate constants. The scheme works well if we consider a first-order kinetics for the formation of $A$, and $B$ and the nucleation of $D_n$, while the growth of phase $D_g$, and the formation of $P$ has to be always catalytic. The corresponding equations for the local transformation degrees has $\alpha_{R,i}$, $\alpha_{A,i}$, $\alpha_{B,i}$, $\alpha_{D,i}$, and $\alpha_{P,i}$ are:

$$\frac{d\alpha_{R,i}}{di} = -k_{RA}\alpha_{R,i}; \tag{5}$$

$$\frac{d\alpha_{A,i}}{di} = -k_{AB}\alpha_{A,i} + k_{RA}\alpha_{R,i}; \tag{6}$$

$$\frac{d\alpha_{B,i}}{di} = k_{AB}\alpha_{A,i} - k_{BD}\alpha_{B,i} - k_{D_nD_g}\alpha_{B,i}\alpha_{D_g,i}; \tag{7}$$

$$\frac{d\alpha_{D,i}}{di} = k_{BD}\alpha_{B,i} + k_{D_nD_g}\alpha_{B,i}\alpha_{D_g,i} - k_{DP}\alpha_{D,i}\alpha_{P,i}; \tag{8}$$

$$\frac{d\alpha_{P,i}}{di} = -k_{DP}\alpha_{D,i}\alpha_{P,i}. \tag{9}$$

We note that the very first impact induces the formation of a certain fraction of $A$ and a smaller fraction of $P$. We use the fraction of $P$ formed at the first impact, $\alpha_{P,start}$, as an adjustable parameter of the model.

The equations describing the global transformation kinetics are:

$$\alpha_R(t) = \sum_{i=1}^{\infty} \chi_i(t)\alpha_{R,i} \quad ; \tag{10}$$

$$\alpha_A(t) = \sum_{i=1}^{\infty} \chi_i(t)\alpha_{A,i} \quad ; \tag{11}$$

$$\alpha_B(t) = \sum_{i=1}^{\infty} \chi_i(t)\alpha_{B,i} \quad ; \tag{12}$$

$$\alpha_D(t) = \sum_{i=1}^{\infty} \chi_i(t)\alpha_{D,i} \quad ; \tag{13}$$

$$\alpha_P(t) = \sum_{i=1}^{\infty} \chi_i(t)\alpha_{P,i} \quad . \tag{14}$$

These equations were used to best fit the experimental data as shown in **Fig.ESI.12.1**

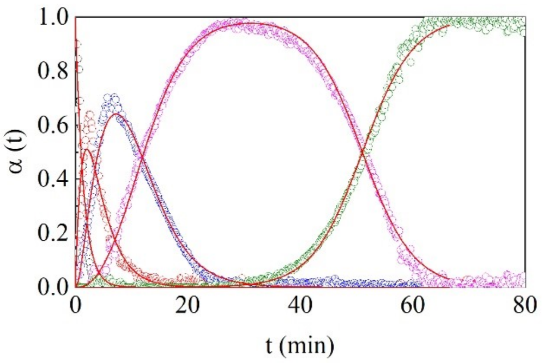


**Fig.ESI.12.1.** Global molar fractions, $\alpha(t)$, obtained with the MCR-ALS model with Kinetic constraint for the 30 μL experiment as a function of the milling time. Best fitting curves are shown.

Fitting parameters are reported in the following table

| $\kappa$ | $k_{RA}$ | $k_{AB}$ | $k_{BD}$ | $k_{D_n D_g}$ | $k_{DP}$ | $\alpha_{P,start}$ |
|---|---|---|---|---|---|---|
| 0.016 | 0.0143 | 0.0068 | 0.001 | 0.0038 | 0.0047 | $1.9 \times 10^{-5}$ |

**60 μL experiment.** In the case of 60 mL kinetics, the conversion of the reactants into the phase $A$ is too fast to be measured experimentally, therefore it is already formed as kinetic monitoring begins. The

simplest kinetic scheme able to account for the experimental curves involves parallel and consecutive reactions as shown below:

$$A \xrightarrow{k_{AB}} B \xrightarrow{k_{BC}} C \xrightarrow{k_{CD}} \left[ D_n \xrightarrow{k_{D_nD_g}} D_g \right] \xrightarrow{k_{DP}} P$$

Here, $k_{AB}$, $k_{BC}$, $k_{CD}$, $k_{D_nD_g}$, and $k_{DP}$ represent apparent rate constants. The scheme works well if we consider a first-order kinetics for the formation of $B$, and $C$ and the nucleation of $D_n$, while the growth of phase $D_g$, and the formation of $P$ has to be always catalytic. The corresponding equations for the local transformation degrees has $\alpha_{A,i}$, $\alpha_{B,i}$, $\alpha_{C,i}$, $\alpha_{D,i}$, and $\alpha_{P,i}$ are:

$$\frac{d\alpha_{A,i}}{di} = - k_{AB}\alpha_{A,i}; \tag{15}$$

$$\frac{d\alpha_{B,i}}{di} = k_{AB}\alpha_{A,i} - k_{BC}\alpha_{B,i}; \tag{16}$$

$$\frac{d\alpha_{B,i}}{di} = k_{AB}\alpha_{A,i} - k_{BC}\alpha_{B,i}; \tag{17}$$

$$\frac{d\alpha_{D,i}}{di} = k_{CD}\alpha_{C,i} + k_{D_nD_g}\alpha_{C,i}\alpha_{D,i} - k_{DP}\alpha_{D,i}\alpha_{P,i}; \tag{18}$$

$$\frac{d\alpha_{P,i}}{di} = - k_{DP}\alpha_{D,i}\alpha_{P,i}. \tag{19}$$

We note that the very first impact induces the formation of a certain fraction of $B$ and a smaller fraction of $P$. We use the fraction of $P$ formed at the first impact, $\alpha_{P,start}$, as an adjustable parameter of the model.

The equations describing the global transformation kinetics are:

$$\alpha_A(t) = \sum_{i=1}^{\infty} \chi_i(t)\alpha_{A,i}; \tag{20}$$

$$\alpha_B(t) = \sum_{i=1}^{\infty} \chi_i(t)\alpha_{B,i}; \tag{21}$$

$$\alpha_C(t) = \sum_{i=1}^{\infty} \chi_i(t)\alpha_{C,i}; \tag{22}$$

$$\alpha_D(t) = \sum_{i=1}^{\infty} \chi_i(t)\alpha_{D,i}; \tag{23}$$

$$\alpha_P(t) = \sum_{i=1}^{\infty} \chi_i(t)\alpha_{P,i} \quad . \qquad (24)$$

These equations were used to best fit the experimental data as shown in **Fig.ESI.5.2.**

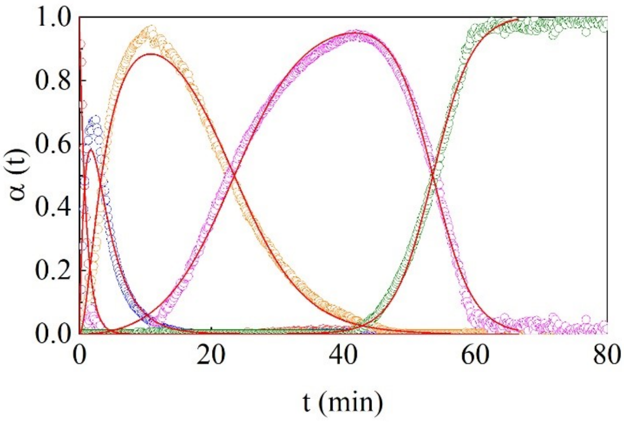


**Fig.ESI.12.2.** Global molar fractions, $\alpha(t)$, obtained with the MCR-ALS model with Kinetic constraint for the 60 μL experiment as a function of the milling time. Best fitting curves are shown.

Fitting parameters are reported in the following table

| $\kappa$ | $k_{AB}$ | $k_{BC}$ | $k_{CD}$ | $k_{D_nD_g}$ | $k_{DP}$ | $\alpha_{P,start}$ |
|---|---|---|---|---|---|---|
| 0.0192 | 0.017 | 0.0055 | 0.0001 | 0.0031 | 0.0059 | $3.4 \times 10^{-5}$ |

**90 μL experiment.** In the case of 90 mL kinetics, the conversion of the reactants into the phase $A$ is too fast to be measured experimentally, therefore it is already formed as kinetic monitoring begins. The simplest kinetic scheme able to account for the experimental curves involves parallel and consecutive reactions as shown below:

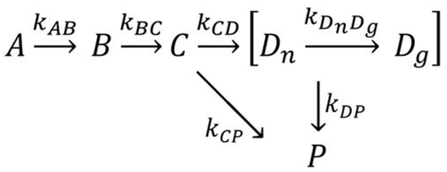


Here, $k_{AB}$, $k_{BC}$, $k_{CD}$, $k_{D_nD_g}$, $k_{DP}$, and $k_{CP}$ represent apparent rate constants. The scheme works well if we consider a first-order kinetics for the formation of $B$, and $C$ and the nucleation of $D_n$, while the growth of phase $D_g$, and the formation of $P$ has to be always catalytic. The corresponding equations for the local transformation degrees has $\alpha_{A,i}$, $\alpha_{B,i}$, $\alpha_{C,i}$, $\alpha_{D,i}$, and $\alpha_{P,i}$ are:

$$\frac{d\alpha_{A,i}}{di} = -k_{AB}\alpha_{A,i};\qquad (25)$$

$$\frac{d\alpha_{B,i}}{di} = k_{AB}\alpha_{A,i} - k_{BC}\alpha_{B,i};\qquad (26)$$

$$\frac{d\alpha_{C,i}}{di} = k_{BC}\alpha_{B,i} - k_{CD}\alpha_{C,i} - k_{D_nD_g}\alpha_{C,i}\alpha_{D,i} - k_{CP}\alpha_{P,i}\alpha_{C,i};\qquad (27)$$

$$\frac{d\alpha_{D,i}}{di} = k_{CD}\alpha_{C,i} + k_{D_nD_g}\alpha_{C,i}\alpha_{D,i} - k_{DP}\alpha_{D,i}\alpha_{P,i};\qquad (28)$$

$$\frac{d\alpha_{P,i}}{di} = -k_{DP}\alpha_{D,i}\alpha_{P,i} + k_{CP}\alpha_{P,i}\alpha_{C,i}.\qquad (29)$$

We note that the very first impact induces the formation of a certain fraction of $B$ and a smaller fraction of $P$. We use the fraction of $P$ formed at the first impact, $\alpha_{P,start}$, as an adjustable parameter of the model.

The equations describing the global transformation kinetics are

$$\alpha_A(t) = \sum_{i=1}^{\infty}\chi_i(t)\alpha_{A,i};\qquad (30)$$

$$\alpha_B(t) = \sum_{i=1}^{\infty}\chi_i(t)\alpha_{B,i};\qquad (31)$$

$$\alpha_C(t) = \sum_{i=1}^{\infty}\chi_i(t)\alpha_{C,i};\qquad (32)$$

$$\alpha_D(t) = \sum_{i=1}^{\infty}\chi_i(t)\alpha_{D,i};\qquad (33)$$

$$\alpha_P(t) = \sum_{i=1}^{\infty}\chi_i(t)\alpha_{P,i};\qquad (34)$$

These equations were used to best fit the experimental data as shown in **Fig.ESI.12.3.**

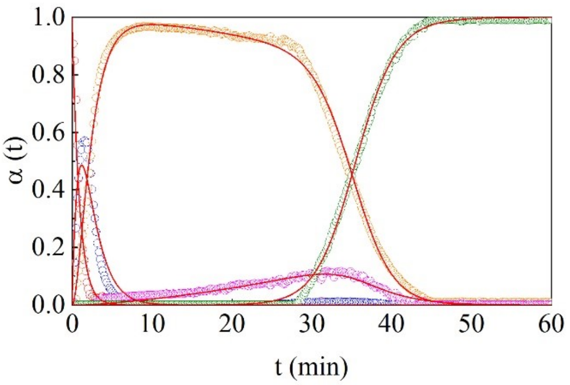


**Fig.ESI.12.3**. Global molar fractions, $\alpha(t)$, obtained with the MCR-ALS model with Kinetic constraint for the 90 µL experiment as a function of the milling time. Best fitting curves are shown.

Fitting parameters are reported in the following table

| $\kappa$ | $k_{AB}$ | $k_{BC}$ | $k_{CD}$ | $k_{D_n D_g}$ | $k_{DP}$ | $k_{CP}$ | $\alpha_{P,start}$ |
|---|---|---|---|---|---|---|---|
| 0.014602 | 0.025506 | 0.013727 | 0.000054 | 0.000963 | 0.006074 | 0.0099 | $1x10^{-5}$ |

**References**


1 A. de Juan, J. Jaumot and R. Tauler, *Anal. Methods*, 2014, **6**, 4964–4976.
2 S. Brown, R. Tauler and B. Walczak, Eds, *Comprehensive chemometrics: chemical and biochemical data analysis*, Elsevier, Amsterdam Oxford Cambridge, MA, Second edition., 2020.
3 A. de Juan, M. Maeder, M. Martı́nez and R. Tauler, *Chemometrics and Intelligent Laboratory Systems*, 2000, **54**, 123–141.
4 J. Saurina, S. Hernández-Cassou, R. Tauler and A. Izquierdo-Ridorsa, *J. Chemometrics*, 1998, **12**, 183–203.
5 T. Andersons, M. Sawall, M. Beese, C. Kubis and K. Neymeyr, *Journal of Chemometrics*, 2024, **38**, e3608.
6 N. Mouton, M. Sliwa, G. Buntinx and C. Ruckebusch, *Journal of Chemometrics*, 2010, **24**, 424–433.
7 D. Pérez-Guaita, G. Quintás, Z. Farhane, R. Tauler and H. J. Byrne, *Cells*, 2022, **11**, 1555.
8 Willem. Windig and Jean. Guilment, *Anal. Chem.*, 1991, **63**, 1425–1432.
9 Travert Arnaud and Fernandez Christian, SpectroChemPy, a framework for processing, analyzing and modeling spectroscopic data for chemistry with Python (version 0.8.1). Zenodo, https://www.spectrochempy.fr.
